# How do postnatal care guidelines in Australia compare to international standards? A scoping review and comparative analysis

**DOI:** 10.1186/s12884-024-06295-4

**Published:** 2024-02-09

**Authors:** Amanda Blair, Annie Tan, Caroline S. E. Homer, Joshua P. Vogel

**Affiliations:** 1 Maternal, Child and Adolescent Health Program, Burnet Institute, 85 Commercial Road, Melbourne, VIC 3004 Australia; 2 Melbourne School of Population and Global Health, The University of Melbourne, 207 Bouverie Street, Carlton, VIC 3053 Australia

**Keywords:** Postnatal care, Postpartum care, Newborn care, Infant feeding, Clinical practice guidelines

## Abstract

**Background:**

There is no single national guideline in Australia on the provision of postnatal care, which means there is potential for significant variation in the standard and quality of care. This review aimed to systematically identify, synthesise, and assess the quality of postnatal care guidelines produced for use in Australia. A second aim was to compare postnatal care recommendations in Australian guidelines to the National Institute for Health and Care Excellence’s (NICE) and the World Health Organization’s (WHO) postnatal care recommendations, to identify gaps and areas of disagreement. We focussed on recommendations regarding postnatal assessment of the woman or newborn, infant feeding, discharge planning, or community-based care.

**Methods:**

A scoping review was undertaken informed by the Preferred Reporting Items for Systematic reviews and Meta-Analyses extension for scoping reviews. A database search and a manual search of state and national government health departments, professional associations and research institute websites was performed to identify relevant guidelines and recommendations. Guideline quality was assessed using the AGREE II tool. Guideline recommendations from Australia were mapped to 67 NICE/WHO recommendations. Recommendations that partially agreed, were modified, or in disagreement underwent further analysis.

**Results:**

A total of 31 Australian postnatal guidelines were identified and overall, these were of moderate- to high-quality. Of the 67 NICE/WHO recommendations, most agreed with the recommendations contained in Australian guidelines. There were five NICE/WHO recommendations with which corresponding Australian recommendations disagreed. There were 12 NICE/WHO recommendations that were commonly modified within Australia’s guidelines. There were three NICE/WHO recommendations that did not appear in any Australian guideline.

**Conclusions:**

Recommendations from postnatal guidelines in Australia have a high level of agreement with corresponding NICE/WHO recommendations. The few disagreements and modifications found in guideline recommendations - both across Australia’s guidelines and between Australia’s and the NICE/WHO guidelines - are worrying and warrant further examination, as they may result in different standards of care across Australia. Identified gaps in guidance should be prioritised for inclusion in new or updated guidelines where appropriate.

**Supplementary Information:**

The online version contains supplementary material available at 10.1186/s12884-024-06295-4.

## Introduction

The postnatal period, commonly defined as the first 6–8 weeks after childbirth [1, 2], is a time of immense change for women and newborns. Women are also at risk of physical and psychological complications as they recover from birth, and learn and adapt to caring for a new infant [3]. Common physical conditions women encounter postnatally include pain from the perineum or the caesarean scar; breast engorgement; constipation; haemorrhoids; urinary or faecal incontinence; back pain and fatigue [4, 5]. Less common, but potentially serious, postnatal complications include infection and postpartum haemorrhage [6]. Postnatal complications can have long-lasting consequences - postpartum depression, anxiety, pelvic organ prolapse, sexual dysfunction and weight retention often affect women throughout the first postnatal year and beyond [6–8]. The early postnatal weeks and months are a particularly critical time in an infant’s life also. Prompt identification and treatment of newborn health conditions in this period can ensure healthy growth and development, promoting better lifelong health [1].

Postnatal care services aim to support a woman’s recovery after birth, provide breastfeeding and parenting education and support, screen for common postnatal conditions, and provide clinical care to women and newborns to enhance their physical and mental wellbeing [9]. Postnatal services must reach all new postnatal women and newborns, be adequately resourced, and be capable of providing evidence-based and respectful care delivered by trained providers. In Australia, postnatal care is provided by multiple healthcare professions - midwives, obstetricians, general practitioners, paediatricians and nurses – across multiple settings, including public or private hospitals, in community-based primary care services, or at home [10]. Ideally, all postnatal care providers and services would use the same clinical practice guidelines, to ensure standardised, equitable and high-quality care is available to all postnatal women and newborns, regardless of where they live or what postnatal services they access.

The Institute of Medicine defines clinical practice guidelines (guidelines) as “statements that include recommendations intended to optimise patient care that are informed by a systematic review of evidence and an assessment of the benefits and harms of alternate care options” [11]. Australia does not currently have a single national guideline for postnatal care, as it has for antenatal care [12]. Instead, Australia’s postnatal care providers and services rely on a mix of different guidance from state and territory government health departments, health organisations, and professional associations.

The World Health Organization (WHO) and the National Institute for Health and Care Excellence (NICE) both recently published updated postnatal care guidelines [1, 2]. The WHO postnatal guideline, which is intended for global use, encourages producers of national, sub-national, and facility level guidelines to adapt the WHO recommendations to fit their local workforce, healthcare system, demographics, and sociocultural expectations [1]. The NICE postnatal guideline is intended for use in the United Kingdom (UK) which, like Australia, delivers maternity care predominantly through the publicly-funded health system. Other contextual similarities between Australia and the United Kingdom, as well as their recency and robust development, mean the NICE postnatal guideline is highly relevant to the Australian context.

We aimed to systematically identify, synthesise, and assess the quality of all postnatal care guidelines that were produced for use in Australia by state and territory governments, professional associations, and health organisations. We also aimed to compare Australia’s postnatal care guideline recommendations to the WHO and NICE recommendations, to identify areas of disagreement, as well as current gaps.

## Methods

### Study design

A scoping review was undertaken. Scoping reviews are ideal when the scope of the available evidence is uncertain, when grey literature forms the majority of material under review, and when research questions extend beyond intervention effectiveness [13, 14]. We followed the Joanna Briggs Institute’s methodological manual for scoping reviews and applied the Preferred Reporting Items for Systematic Reviews and Meta-Analyses extension for scoping reviews (PRISMA-ScR) checklist [13, 15] (Supplementary file 1 - PRISMA checklist). The review protocol was published freely online [16].

### Eligibility criteria

The eligibility criteria are outlined in Table 1. Only documents that met the Institute of Medicine’s operational definition of a clinical guideline [11] were considered eligible. That is, they had to include recommendations informed by a systematic review of evidence. To be eligible, guidelines had to be intended for use in Australia and include recommendations on one or more of the following aspects of postnatal care: assessment of the woman or the newborn; infant feeding; discharge planning; or community-based care. As definitions of the postnatal period can differ greatly, we adopted the NICE definition, meaning guidelines were eligible if they contained recommendations related to the first eight weeks after birth [2]. Guidelines that focussed only on treatment of specific postnatal complications, including care specific to preterm infants, were outside the scope of this review. The search was restricted to guidelines published in English. Guidelines published from 2010 onwards were considered eligible. While this extends beyond the recommended 3–5 year updating period for guidelines [17], this frequency of updates is not always adhered to. By including guidelines published from 2010 onwards, we hoped to understand how current all guidelines are that are likely to still be in use by Australia’s maternity providers.

**Table 1:** PICOST criteria for inclusion and exclusion

Criteria	Inclusion	Exclusion
**Population/** **concept**	Postnatal care guideline relating to any one or more of: - Assessment of healthy, low-risk mothers - Assessment of healthy, low-risk newborns - Infant feeding - Discharge planning - Community-based postnatal care	- Guideline on preconception, antenatal and/or intrapartum care only - Relates to care of mothers or newborns commencing after the first 8 weeks after birth. - Assessment of high-risk women and newborns (including care specific to pre-term infants born before 37 weeks of gestation), and treatment of specific postnatal complications.
**Intervention/** **context**	- Australian postnatal care guidelines aimed at a national, state, or territory level.	- Facility level guidelines.
**Outcomes**	- Clinical or health system recommendations	N/A
**Sources**	Documents that meet the Institute of Medicine’s definition of a clinical guideline: - Includes recommendations designed to enhance patient care - Informed by a systematic review of evidence	- Research articles (Narrative reviews, systematic reviews, randomised controlled trials, observational studies, case studies, qualitative studies, program evaluations) - Commentary pieces - Editorials - Policy documents without clinical recommendations
**Timeframe**	- Published in or after the year 2010	- Published before 2010 and after the search date (Final search conducted 10th July 2023) - Previous editions of guidelines included in the review
**Additional criteria**	- Available in English	- Guidelines endorsed by but not created by Australian governments or professional bodies of interest

### Search strategy and screening

A manual search of websites likely to contain postnatal clinical guidelines was conducted between 26 July and 2 August 2022. This included searching the websites of all state, territory and federal Government health departments, national research institutes, and relevant professional associations such as the Australian College of Midwives (ACM), and The Royal Australian and New Zealand College of Obstetricians and Gynaecologists (RANZCOG) (See Supplementary File 2 for a full list of websites searched). Where websites had simple search function capabilities the following search terms were used: postnatal, postpartum, perinatal, maternal, newborn, neonatal, infant, and breastfeeding. In addition, we systematically searched the Trip Pro database on 10 July 2023 using the same key terms, filtering for clinical guidelines in Australia (see Supplementary file 3 for search strategy).

Guideline selection involved two reviewers (AB and AT) independently screening titles and then full texts of guidelines using Covidence according to the eligibility criteria. Conflicts at either stage of screening were discussed between the two reviewers and if a consensus could not be reached a third reviewer (JPV or CSEH) was consulted. Reference lists of included guidelines were also searched to find other potentially relevant guidelines.

### Quality appraisal

Quality of identified Australian guidelines was independently assessed by two reviewers (AB and AT) using the Appraisal of Guidelines for Research and Evaluation (AGREE) II tool [18]. The AGREE II tool comprises 23 items over six domains: scope and purpose, stakeholder involvement, rigour of development, clarity of presentation, applicability, and editorial independence. Each item was rated on a scale of one to seven. Item scores that differed by more than two between the reviewers were discussed and alterations to scores made where appropriate. Domain scores were then calculated according to the AGREE II user manual [18].

### Data extraction

Data were extracted independently by two reviewers (AB and AT) into a pre-designed Excel spreadsheet. Conflicts at the data extraction phase were resolved during discussion between the two reviewers or with the assistance of CSEH or JPV. Extracted data included guideline characteristics (publisher, publication year, funder, and location for guideline use), as well as the data on the recommendations.

First, we extracted the 73 individual recommendations from the NICE and WHO postnatal guidelines pertaining to our five areas of interest. These recommendations were organised into distinct care practices, allowing us to connect similar (or related) NICE and WHO recommendations into a single practice recommendation. This resulted in 67 distinct recommendations, the details of which can be found in Supplementary File 4. Ten recommendations relate to assessment of the women (# 1–10), 20 relate to assessment of the newborn (# 11–30), 22 are about infant feeding (# 31–52), ten are about discharge planning (# 53–62), and five are about community-based care (# 63–67).

From the 31 Australian guidelines identified, we determined whether and how many guidelines had recommendations pertaining to the 67 practices recommended by NICE/WHO. We compared each Australian recommendation to the corresponding NICE/WHO recommendations and classified it as ‘agreed with recommendation’; ‘partially agreed’; was a ‘modification of the recommendation’; and ‘disagreed’. We also identified those Australian guidelines where a NICE/WHO recommendation was absent, and whether it was within the scope of a guideline or not (see Table 2 for further explanation of all categories).

**Table 2:** Mapping guideline recommendations – classification system

Classification	Definition
**Agree**	The Australian recommendation agrees with the NICE/WHO recommendation of interest.
**Partially agree**	The Australian recommendation does not contain all components of the individual NICE/WHO recommendation, but there is agreement with some components.
**Modification of recommendation**	The Australian recommendation uses a substantively different approach to solve the same health problem addressed in the NICE/WHO recommendation, to achieve a similar health outcome. For example, recommendations may be used in a different healthcare setting (community services rather than the hospital) or may be applied to a sub-population only.
**Disagree**	The Australian recommendation disagrees with the NICE/WHO recommendation and is likely to result in a different health or quality of care outcome.
**Absent**	The NICE/WHO recommendation does not appear in an Australian guideline, even though the NICE/WHO recommendation would be considered within scope of the Australian guideline.
**Recommendation not in scope of guideline**	The NICE/WHO recommendation does not appear in an Australian guideline, however the recommendation would be considered out of scope of the Australian guideline. This may be due to the NICE/WHO recommendation falling outside the subject area or healthcare setting of the Australian guideline.

### Data analysis

For each of the 67 NICE/WHO recommendations, we tabulated the number of Australian guidelines that agreed with the recommendations, partially agreed, those that presented a modification of the recommendation, disagreed with the recommendation, where the recommendation was absent, and where the recommendation was not in scope of the guideline. Using this, we identified whether Australian recommendations were in agreement (or not) with NICE/WHO recommendations. It also allowed us to identify recommendations that were absent from Australian guidelines.

When Australian recommendations were classified as partially agree, modified, or disagree, we performed content analysis [19]. Verbatim text was extracted from the Australian guidelines and imported into NVivo where we organised data, and summarised all partial agreements, modifications, and disagreements. This analysis was undertaken by AB and the results cross-checked by a second reviewer (either AT, CSEH, or JPV), with minor amendments made. Where we identified recommendations from Australian guidelines that did not appear in NICE/WHO guidelines, these were imported verbatim into NVivo for content analysis. Any recommendation that appeared in four or more of Australia’s guidelines were reported as an ‘additional recommendation’. Additional recommendations are reported in detail in Supplementary File 4 (#A1-A13).

## Results

### Characteristics of included Australian guidelines

**Fig. 1:**
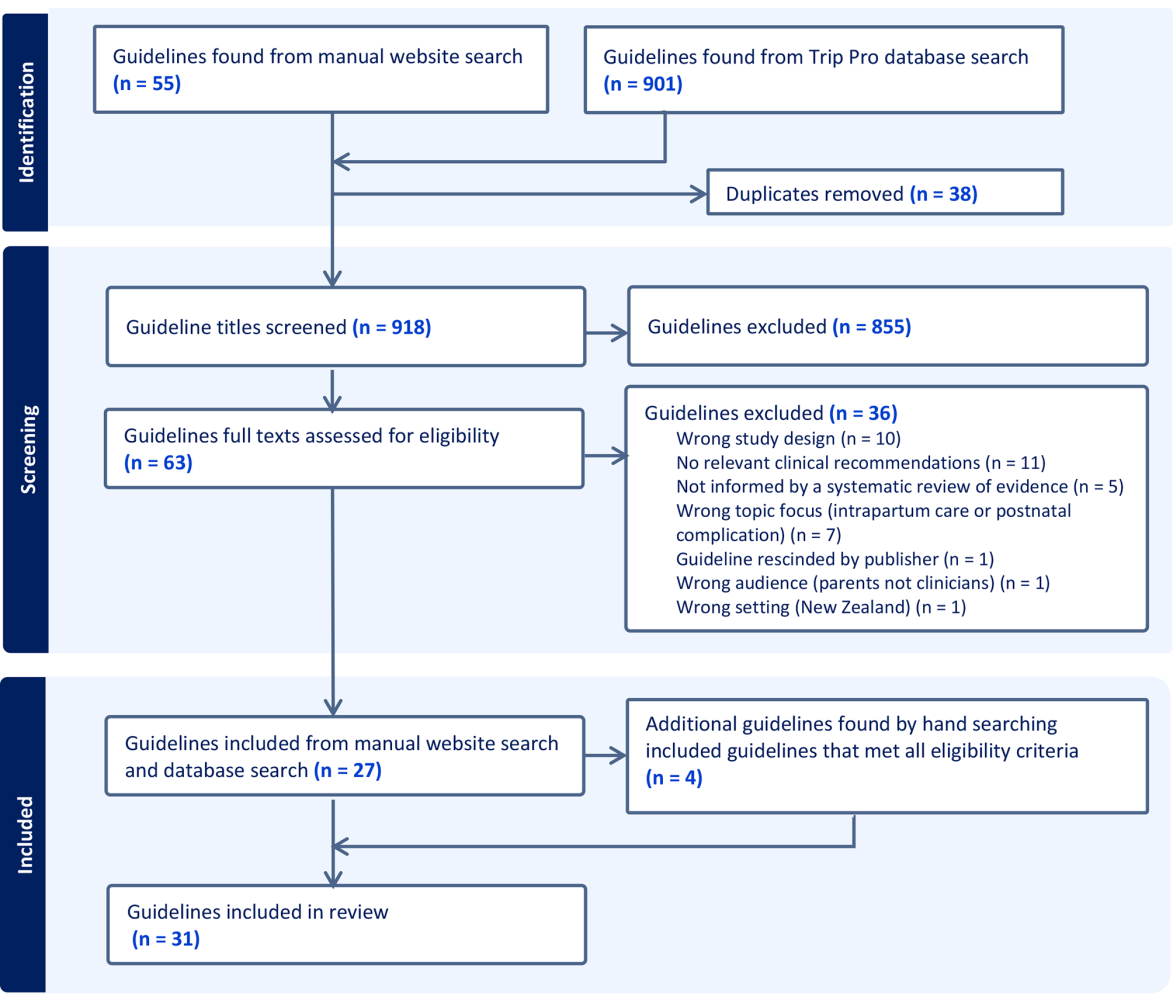
PRISMA flow diagram

We identified 918 potentially eligible guidelines, of which 27 were included. Four additional guidelines were found during hand searching, producing 31 guidelines in total (Fig. 1). Of these, 21 were published by state governments (11 in South Australia [20–30], six in Queensland [31–36], three in Victoria [37–39], and one in Western Australia [40]), four were published by the Australian federal government [41–44], three by professional bodies [45–47], two by health organisations [48, 49], and one by a combined professional body and community health organisation [50].

The focus and scope of guidelines varied. Nine of the guidelines were intended for use by a specific health worker cadre, such as maternal and child health nurses [37–39, 44], general practitioners [30, 36, 47, 50], or midwives [46]. For 25 guidelines, the scope was defined by a particular aspect of postnatal care, the most common being infant feeding (*N* = 6 guidelines), perinatal mental health (*N* = 5 guidelines), perineal care (*N* = 3 guidelines), and newborn hearing screening (*N* = 3 guidelines). Only one guideline covered postnatal care more broadly [20]. Of the 31 guidelines, 26 were created or updated in the five years prior to the search (i.e. produced since August 2017). Table 3 summarises characteristics of included guidelines.

**Table 3:** Australian guideline characteristics and quality appraisal scores

Guideline characteristics				AGREE II quality appraisal scores						
Guideline name	Year of publication	Publisher (category)	Location for use	Domain 1. Scope and purpose	Domain 2. Stakeholder involvement	Domain 3. Rigour of development	Domain 4. Clarity of presentation	Domain 5. Applicability	Domain 6. Editorial independence	Recommended for use?
**General postnatal care guidelines**										
Postnatal Care: Routine care of the well woman and neonate (20)	2021	SA Health, SA Maternal, Neonatal & Gynaecology Community of Practice (state government)	South Australia	86.11	36.11	22.92	80.56	31.25	0.00	Yes, with modifications
**Maternal physical health guidelines**										
Perineal Care and Repair (21)	2021	SA Health, SA Maternal, Neonatal & Gynaecology Community of Practice (state government)	South Australia	83.33	44.44	35.42	86.11	25.00	4.17	Yes, with modifications
Perineal care (31)	2020	Queensland Clinical Guidelines, Queensland Health (state government)	Queensland	91.67	69.44	77.08	94.44	68.75	62.50	Yes
Third and fourth degree tear management (22)	2018	SA Health, SA Maternal, Neonatal & Gynaecology Community of Practice (state government)	South Australia	80.56	27.78	12.50	66.67	20.83	0.00	Yes, with modifications
Bladder management for intrapartum and postnatal women (23)	2018	SA Health, SA Maternal, Neonatal & Gynaecology Community of Practice (state government)	South Australia	80.56	36.11	12.50	91.67	33.33	0.00	Yes, with modifications
**Perinatal mental health guidelines**										
Mental Health Care in the Perinatal Period: Australian Clinical Practice Guideline (48)	2023	The Centre of Perinatal Excellence (health organisation)	Australia (national)	100.00	100.00	96.88	100.00	85.42	100.00	Yes
Best Practice Statement: Mental Health Care in the Perinatal Period (45)	2021	Royal Australian and New Zealand College of Obstetricians and Gynaecologists (professional body)	Australia and New Zealand	91.67	44.44	51.04	83.33	58.33	62.50	Yes, with modifications
Anxiety and Depression in the Perinatal Period (24)	2019	SA Health, SA Maternal, Neonatal & Gynaecology Community of Practice (state government)	South Australia	80.56	36.11	15.63	88.89	35.42	0.00	Yes, with modifications
Perinatal mental health and psychosocial assessment (37)	2019	Victorian Government, Department of Health and Human Services (state government)	Victoria	77.78	50.00	22.92	86.11	64.58	0.00	Yes, with modifications
Perinatal and Infant Mental Health Model of Care – a framework (40)	2016	Government of Western Australia, Department of Health (state government)	Western Australia	86.11	91.67	37.50	91.67	62.50	0.00	Yes, with modifications
**Infant health guidelines**										
Newborn Hearing Screening (25)	2022	SA Health, SA Maternal, Neonatal & Gynaecology Community of Practice (state government)	South Australia	88.89	38.89	22.92	80.56	83.33	0.00	Yes, with modifications
Neonatal Jaundice (26)	2021	SA Health, SA Maternal, Neonatal & Gynaecology Community of Practice (state government)	South Australia	86.11	41.67	21.88	94.44	43.75	0.00	Yes, with modifications
Newborn baby assessment (routine) (32)	2021	Queensland Clinical Guidelines, Queensland Health (state government)	Queensland	75.00	55.56	54.17	83.33	54.17	66.67	Yes
Neonatal jaundice (33)	2019	Queensland Clinical Guidelines, Queensland Health (state government)	Queensland	97.22	91.67	76.04	88.89	62.50	45.83	Yes
Audiology Diagnostic Assessment Protocol (34)	2018	Healthy Hearing Program’s Audiology Working Group, Children’s Health Queensland (state government)	Queensland	88.89	50.00	22.92	83.33	52.08	0.00	Yes, with modifications
Neonatal Hip Screening and Management of Developmental Dysplasia of the Hip (27)	2017	SA Health, SA Maternal, Neonatal & Gynaecology Community of Practice (state government)	South Australia	86.11	41.67	27.08	72.22	43.75	0.00	Yes, with modifications
National Framework for Neonatal Hearing Screening (41)	2013	Australian Government (federal government)	Australia (national)	94.44	63.89	26.04	88.89	64.58	0.00	Yes, with modifications
**Infant feeding guidelines**										
BFHI Australia: Maternity Facility Handbook (revised 2021) (49)	2021	Baby Friendly Health Initiative, Australia (health organisation)	Australia (national)	97.22	25.00	33.33	97.22	68.75	0.00	Yes
Establishing breastfeeding (35)	2021	Queensland Clinical Guidelines, Queensland Health (state government)	Queensland	94.44	66.67	75.00	94.44	68.75	66.67	Yes
Breastfeeding (28)	2019	SA Health, SA Maternal, Neonatal & Gynaecology Community of Practice (state government)	South Australia	69.44	27.78	26.04	86.11	29.17	0.00	Yes, with modifications
Expressed breast milk safe management and administration in SA 2018 (29)	2018	SA Health, SA Maternal, Neonatal & Gynaecology Community of Practice (state government)	South Australia	69.44	66.67	16.67	75.00	41.67	0.00	Yes, with modifications
Australian Dietary Guidelines (42)	2013	Australian Government, National Health and Medical Research Council, and the Department of Health and Ageing (federal government)	Australia (national)	97.22	97.22	90.63	94.44	58.33	62.50	Yes
Infant Feeding Guidelines Information for Health Workers (43)	2012	Australian Government, National Health and Medical Research Council, and the Department of Health and Ageing (federal government)	Australia (national)	100.00	94.44	84.38	100.00	50.00	54.17	Yes, with modifications
**Maternal and child health guidelines**										
Maternal and child health program standards (38)	2019	Victorian Government, Department of Health and Human Services (state government)	Victoria	91.67	86.11	18.75	66.67	50.00	0.00	Yes, with modifications
Maternal and child health service practice guidelines 2009 (reissued 2019) (39)	2019	Victorian Government, Department of Health and Human Services (state government)	Victoria	61.11	13.89	14.58	80.56	33.33	0.00	Yes, with modifications
National Framework for Universal Child and Family Health Services (44)	2011	National Child Health and Wellbeing subcommittee of the Australian Population Health Development Principal Committee of the Australian Health Ministers’ Conference (federal government)	Australia (national)	72.22	75.00	28.13	33.33	62.50	0.00	Yes, with modifications
**Midwifery guidelines**										
National midwifery guidelines for consultation and referral (46)	2021	Australian College of Midwives (professional body)	Australia (national)	88.89	77.78	28.13	91.67	35.42	0.00	Yes, with modifications
**General practitioner and shared care guidelines**										
Guidelines for preventive activities in general practice (47)	2021	Royal Australian College of General Practitioners (professional body)	Australia (national)	88.89	63.89	56.25	69.44	52.08	45.83	Yes, with modifications
Maternity Shared Care Operational Framework (36)	2021	Queensland Clinical Guidelines, Queensland Health (state government)	Queensland	100.00	94.44	57.29	58.33	79.17	66.67	Yes, with modifications
South Australian GP Obstetric Shared Care Protocols - Clinical Directive (30)	2020	SA Health, SA Maternal, Neonatal & Gynaecology Community of Practice (state government)	South Australia	83.33	61.11	14.58	63.89	27.08	0.00	Yes, with modifications
National guide to a preventive health assessment for Aboriginal and Torres Strait Islander people (50)	2018	National Aboriginal Community Controlled Health Organisation, and The Royal Australian College of General Practitioners (community health organisation in partnership with a professional body)	Australia (national)	97.22	61.11	81.25	86.11	60.42	100.00	Yes, with modifications
**Average AGREE II domain scores across guidelines**				86.56	59.05	40.63	82.53	51.81	23.79	

### Quality appraisal of Australian guidelines

Included guidelines were of moderate to high quality (Table 3). Of the six AGREE II domains, the highest scores were achieved for domain 1: scope and purpose (86.6%), and domain 4: clarity of presentation (82.5%). The lowest score was for domain 6: editorial independence (23.7%), with very few guidelines reporting the role of the funding body in guideline development or competing interests of guideline developers (Supplementary file 5).

### NICE/WHO recommendations compared to Australian guidelines

Recommendations in Australian guidelines mostly agreed with their corresponding NICE/WHO recommendations. There were five recommendations (# 3, 22, 27, 31, and 46) with which an Australian guideline disagreed. Disagreements were in relation to universal screening of emotional wellbeing, frequency of newborn growth assessments, universal TcB screening, exclusive breastfeeding duration, and vitamin D supplementation for breastfeeding women. There were 12 NICE/WHO-recommendations, where the corresponding recommendations in Australian guidelines were a modified version. These modifications usually related to the practice being applied in a different setting (community rather than the health facility), or related to different timing for postnatal contacts and assessments. Three NICE/WHO recommendations (# 8, 18, and 66) – about the use of perineal pain scales, absence of fever in some newborn infections, and indications for an additional postnatal home visit - did not appear in any Australian guideline. A total of 13 practices (#A1- A13) were commonly recommended in Australian guidelines that were additional to the recommendations included in the NICE and/or WHO guidelines.

### Recommendations: Assessment of the woman

Of the 10 NICE/WHO recommendations relating to assessment of women, five recommendations (# 1, 2, 5, 7, and 9) appeared frequently in Australian guidelines (Table 4 and Supplementary File 4). However, few Australian guidelines contained these recommended practices in their entirety, each missing one or more components of a recommendation. For example, in recommendation 5 pertaining to physical assessments after 24 h, none of the partially agreeing guidelines recommended assessing for signs and symptoms of thromboembolism, anaemia, and pre-eclampsia, while eight guidelines recommended assessing bladder and bowel function [21–23, 30, 31, 38, 39, 47].

**Table 4:** Assessment of the woman and newborn – comparison and level of agreement between NICE/WHO recommendations and Australian postnatal guidelines

Recommendations	Number of Australian guidelines mapped to each category					
	Agree	Partially agree	Modified	Disagree	Absent	Not in scope
**Assessment of the woman**						
1. Information provision: general	3	19	0	0	0	9
2. Information provision: signs for care seeking	1	6	1	0	2	21
3. Assessing emotional wellbeing	11	2	2	1	0	15
4. Physical assessments in the first 24 h	1	2	0	0	0	28
5. Physical assessments after the first 24 h	2	10	0	0	1	18
6. Vaginal bleeding assessment and PPH risk factors	1	1	2	0	3	24
7. Assessing perineal wound healing	2	6	1	0	1	21
8. Validated pain scale for measuring perineal pain	0	0	0	0	10	21
9. Responding to women’s concerns about perineal healing	4	3	1	0	2	21
10. Assessment at 6–8 weeks after birth	4	6	6	0	0	15
**Assessment of the newborn**						
11. Parental concerns and reviewing history	7	11	0	0	1	12
12. Red flags for serious illness	1	9	3	0	1	17
13. Significance of a change in behaviour/symptoms	7	3	0	0	2	19
14. Actions after identification of red flags	5	3	1	0	3	19
15. Information provision to parents: red flags and care seeking	5	1	0	0	4	21
16. Actions when meconium not passed in the first 24 h	3	1	3	0	0	24
17. Identification and management of neonatal infection	1	4	0	0	4	22
18. Fever not always present with neonatal infection	0	0	0	0	9	22
19. Complete physical assessment	4	9	0	0	0	18
20. Identifying serious illness: clinical patterns	2	1	2	0	6	20
21. Developmental assessment at 6–8 weeks	2	4	0	0	2	23
22. Weight and head circumference measurements	4	4	4	2	0	17
23. Growth faltering identification and management	2	11	0	0	1	17
24. Newborn bloodspot screening	4	0	0	0	1	26
25. Universal newborn hearing screening	8	3	2	0	1	17
26. Universal newborn screening for abnormalities of the eye	5	2	0	0	2	22
27. Universal screening for hyperbilirubinaemia with TcB at discharge	0	0	2	2	1	26
28. Insufficient evidence for or against universal screening with TSB at discharge	3	0	0	0	2	26
29. Responding to parental concerns	6	4	1	0	3	17
30. Communicating with parents using the Baby Check scoring system	0	0	4	0	7	20

Assessing women’s emotional wellbeing (recommendation # 3) was well covered and in agreement or partial agreement with 13 Australian guidelines. However, the *National guide to a preventive health assessment for Aboriginal and Torres Strait Islander people* guideline recommended against universal screening for depression (as suggested by NICE and WHO), but did recommend assessing emotional well-being in women who are experiencing or at risk of family violence [50]. Another example, recommendation 10, advises that a GP assesses a woman 6 to 8 weeks after birth. In Australia, four guidelines recommended that the assessment be conducted by a provider other than a GP (e.g. maternal and child health nurse or obstetrician) [38, 39, 44, 45], and two guidelines recommended alternate timings/frequency of assessments, such as at two and six weeks [20, 23]. Use of a validated pain scale for measuring perineal pain is recommended by NICE (recommendation 8) but did not appear in the WHO guideline or any Australian guideline.

We found eight recommendations (# A1-A8) that related to maternal health assessment that were commonly found in Australian guidelines that were not present in the NICE or WHO guidelines (Supplementary file 4). These related to perinatal mental health, including recommendations to evaluate parent-infant interactions (# A2), language and cultural appropriateness of mental health screening tools for Indigenous Australian women and for women from migrant and refugee populations (# A4). Others highlighted the need for ongoing clinician training to enable them to competently assess and treat women using a woman-centred approach (# A7 and A8).

### Recommendations: Assessment of the newborn

Of the 20 NICE/WHO recommendations (# 11–30) relating to assessment of the newborn, one did not appear in any Australian guidelines (Table 4, Supplementary file 4). This was NICE recommendation 18 that advises clinicians to be aware that fever may not be present in young babies with a serious infection.

NICE recommends a newborn’s weight and head circumference be measured in the first week of life and at around eight weeks, and only at other times if there are concerns. Two Australian guidelines disagreed, recommending measurements occur at every appointment, noting that reviewing growth charts with parents can incentivise attendance at appointments [44, 47]. Four other guidelines presented alternate timings or different reasons for performing measurements [39, 43, 46, 50]. Two Australian guidelines disagreed with universal screening for neonatal hyperbilirubinaemia by transcutaneous bilirubinometer (TcB) (recommendation # 27), recommending that TcB measurement be performed only in certain clinical scenarios [32, 33].

NICE/WHO recommendation 12 presents the “red flags” clinicians should assess for as possible indicators of serious illness in the newborn. Among others these “red flags” included: pale or blue appearance, unresponsiveness, a weak high-pitched cry, and frequent forceful vomiting. Multiple Australian guidelines had additional “red flags”, including infrequent/scant stools or urination, failure to gain weight and excessive weight loss [35, 43, 46]. Another guideline recommended maintaining a high level of clinical suspicion for infants with risk factors of fetal alcohol syndrome, microcephaly, or convulsions [50].

The NICE guideline recommends that clinicians be aware that fever may not be present in young infants with a serious infection (# 18). This recommendation did not appear in any Australian guidelines, or in the WHO guideline. The NICE guideline also recommends the use of the Baby Check Scoring system to supplement clinical assessments and to aid parents in describing their child’s condition (# 30). This scoring system was developed in the UK and was not included in any Australian guidelines. Four Australian guidelines recommended alternate tools to elicit parental concerns about an infant’s health and development, including the Parents’ Evaluation of Developmental Status (PEDS) [38, 39, 47], Ages and Stages Questionnaire [47], and using parent report questionnaires in the patient-held record [50].

### Recommendations: infant feeding

Australian guidelines contained, and agreed with, all but one of the 22 NICE/WHO infant feeding recommendations (# 31–52) (Table 5). NICE recommends vitamin D supplements for all breastfeeding women (# 46). Two Australian guidelines disagreed - one that vitamin D supplementation is not routinely required [28], while the other stated supplementation should be limited to at-risk (dark skinned and veiled) women [43]. A WHO recommendation (# 31) states all babies should be exclusively breastfed from birth until six months of age. Five Australian guidelines agreed [28, 35, 42, 43, 49], though the Australian *Guidelines for preventive activities in general practice* encourage exclusive breastfeeding until 4–6 months of age [47], while the *National guide to a preventive health assessment for Aboriginal and Torres Strait Islander people* recommend a range of durations from three to six months, depending on the clinical indications and health goals [50]. NICE does not indicate an exclusive breastfeeding duration, though states women should be informed of the benefits of breastfeeding even if only done for a short time [2].

**Table 5:** Infant feeding – comparison and level of agreement between NICE/WHO recommendations and Australian postnatal guidelines

Recommendations	Number of Australian guidelines mapped to each category					
	Agree	Partially agree	Modified	Disagree	Absent	Not in scope
**Infant feeding**						
31. Exclusive breastfeeding until 6 months	5	4	1	1	2	18
32. Facility breastfeeding policies	5	1	1	0	4	20
33. Provider breastfeeding knowledge and skills	5	6	1	0	3	16
34. General principles for providing breastfeeding support (e.g. privacy, respect, consent)	0	7	1	0	5	18
35. Discussing infant feeding holistically	5	5	0	0	4	17
36. Information provision: benefits of breastfeeding	4	11	0	0	1	15
37. Information provision formats: in person and in written/digital formats	2	10	0	0	0	19
38. In person support until breastfeeding established	5	2	0	0	4	20
39. Populations that may require extra support to start and continue breastfeeding	3	1	3	0	4	20
40. Detailed information provision (responsive feeding, breastfeeding positions, expressing, complications)	0	18	0	0	1	12
41. Information provision to partners: how to support a breastfeeding woman	3	3	1	0	3	21
42. Encouraging early skin-to-skin contact	6	1	1	0	4	19
43. Assessing breastfeeding and addressing concerns	6	3	0	0	2	20
44. Components of breastfeeding assessment	3	9	0	0	4	15
45. Actions when ongoing breastfeeding concerns	3	10	0	0	1	17
46. Vitamin D supplementation	0	0	0	2	9	20
47. Right to breastfeed in public spaces	2	0	1	0	6	22
48. Discussing feeding options with parents considering formula feeding	4	0	1	0	4	22
49. Detailed information provision about formula (first infant formula, sterilisation, supplementing breastfeeding)	2	4	0	0	3	22
50. Provision of formula information: individual, in person and written/digital formats	2	4	0	0	3	22
51. Supporting formula feeding: observing feed, recognising and responding to feeding cues, bonding with baby	2	3	0	0	5	21
52. Supporting parents to make informed decisions about infant feeding	5	0	0	0	4	22

An additional five infant feeding recommendations (# A9-A13) were found in Australian guidelines that were not present as NICE or WHO guideline recommendations (Supplementary File 4). These encouraged rooming-in (# A9), discouraged pacifier and bottle use in breastfed infants in the first 4–6 weeks of life (# A10), encouraged iodine supplementation in all breastfeeding women (# A11), and encouraged clinician awareness of the rare but important indications to discourage women from breastfeeding (# A13).

### Recommendations: discharge planning

Of the ten NICE/WHO recommendations related to discharge planning (# 53–62), five (# 55, 57, 58, 60, 62) were commonly present and in agreement with Australian guidelines (Table 6). The NICE/WHO recommendation for a 24 h minimum length of stay in health facilities after vaginal births (# 54) was in disagreement with two Australian guidelines; one recommended discharge within 24 h is possible provided that parents are advised of the neonatal signs and symptoms that warrant urgent medical attention [32], while the other stated if the woman and baby have no complications discharge is possible within 6 h of birth (for a vaginal birth) or within 24 h (for a caesarean birth) [20].

**Table 6:** Discharge planning and community-based care – comparison and level of agreement between NICE/WHO recommendations and Australian postnatal guidelines

Recommendations	Number of Australian guidelines mapped to each category					
	Agree	Partially agree	Modified	Disagree	Absent	Not in scope
**Discharge planning**						
53. Minimum of four postnatal contacts	1	3	5	0	0	22
54. Care in health facility for 24 h	0	0	1	2	2	26
55. Physical assessments before discharge	1	12	1	0	7	10
56. Assessing skills to care for self (woman) and infant (parents) before discharge	2	11	6	0	6	6
57. Communicating transfer of care between providers and with parents	9	2	2	0	3	15
58. What providers should communicate at transfer of care	5	18	0	0	7	1
59. Discussing timing of discharge with women	3	3	4	0	1	20
60. Considerations for deciding the time of discharge	4	4	1	0	1	21
61. Information provision prior to discharge including care-seeking behaviours	1	18	9	0	1	2
62. Contraception information and services	6	0	0	0	6	19
**Community-based care**						
63. First contact after home birth	1	2	1	0	3	24
64. Timing of community contacts	1	9	6	0	1	14
65. Home visits in the first week	1	10	2	0	2	16
66. Considerations for an additional early home visit	0	0	0	0	7	24
67. Psychological/psychosocial interventions for preventing depression and anxiety	13	1	1	0	0	16

The WHO guideline recommends a minimum of four postnatal contacts (# 53). This recommendation was included in four Australian guidelines, with a further five recommending other numbers of contacts in specific circumstances. For example, the *Perinatal and Infant Mental Health Model of Care – a framework* recommended all women have their psychological health assessed at an early postnatal home visit and at a six-week appointment in the community (two postnatal contacts) [40]. Women with symptoms or at high risk of poor mental health should have additional contacts [40].

The NICE and WHO guidelines recommend that, prior to discharge, providers should assess the woman’s confidence to care for herself and the parent’s skills to care for the newborn, discussing the timing of discharge with the woman, and providing key postnatal information to parents (# 56, 59 and 61). Numerous Australian guidelines agreed, however there were modifications as well. Most modifications related to recommending these care practices take place within community health care settings rather than at discharge from the birthing facility. For example, three guidelines recommend discussing with women and families the timing of transfer between two community-based health services [34, 37, 50], highlighting the importance of providing discharge planning guidance in different maternity settings.

### Recommendations: community-based care

Australian guidelines agreed or partially agreed with three (# 63, 65, 67) of the five NICE/WHO recommendations on community-based care (# 63–67) (Table 6). Only one, *Postnatal Care: Routine care of the well woman and neonate*, used the same timeframes recommended by NICE/WHO for the first postnatal contact at home after a home birth (within 24 h of birth) or after discharge from the birthing facility (within 36 h of discharge) [20].

The NICE/WHO-recommended timeframes for subsequent postnatal contacts in the community (48–72 h, 7–14 days, and during week six after birth) (# 64) differed from six Australian guidelines which use different frequencies. Three guidelines recommended a home visit in the first week, followed by appointments in the community at two, four and eight weeks [37–39]. One guideline recommended three postnatal contacts for newborns including a full examination within 48 h of birth and follow up assessments at 5–7 days and at 6 weeks of age [32]. Another guideline recommended a newborn’s hips be assessed four times – once in the hospital or after home birth, then again in the community at 1–4 weeks, 6–8 weeks, and 6–9 months [27]. Finally, one guideline recommended newborns with jaundice require individualised, structured follow up in the community [26].

NICE recommends an additional early postnatal home visit for women who did not receive an antenatal home visit with a midwife/health visitor (# 66) - this was not in any Australian guideline, though antenatal home visits are not common in Australia.

## Discussion

### Summary of findings and implications

This review identified 31 Australian postnatal care guidelines, published by a mix of Australian state and territory governments, the federal government, health organisations, and professional organisations. This is significantly more than the two Australian postnatal guidelines identified in a 2014 review by Haran and colleagues [51]. Thirty Australian guidelines pertained to a single aspect of postnatal care or were aimed at a specific health worker cadre. This means that health workers providing postnatal care in Australia must search for and read through multiple guidelines to deliver evidence-based care, some of which disagree. Consolidating these recommendations into a single, navigable, and updatable resource could ease the burden of “guideline overload” that Australian clinicians currently face [52]. The Living Evidence for Australian Pregnancy and Postnatal care (LEAPP) Guidelines project, funded by the Australian Government Department of Health and Aged Care, will undertake this consolidation of recommendations as it creates Australia’s first national postnatal guideline [53]. This review forms part of the planning and prioritisation process for these forthcoming national guidelines.

Twenty-six Australian guidelines were created or updated within the last five years, suggesting they are reasonably up to date [54]. AGREE II assessments ranged from moderate- to high-quality which is also encouraging. Common areas for improvement were lack of detail on guideline development process, and lack of reporting on editorial independence. Normalising the use of tools such as AGREE II and the National Health and Medical Research Council’s Standards for Guidelines would yield improvements in guideline production and reporting [18, 54].

Our comparative analysis revealed high agreement between the recommendations contained in Australian, NICE, and WHO guidelines. Across the 67 NICE/WHO recommendations, only five were in disagreement with recommendations contained in one or more Australian guidelines. Only three recommendations did not appear in a single Australian guideline (# 8, 18, 66). Recommendation 66 from the NICE guideline was specific to the United Kingdom’s models of maternity care (antenatal home visits), while recommendations 8 and 18 (about the use of perineal pain scales and the absence of fever in some newborn infections), would be relevant to the Australian and international contexts. We identified many examples where Australian recommendations constituted a modified version of NICE/WHO recommended practices. The benefits of modifying or adapting recommendations to fit local contextual factors has been well established [52, 55, 56]. Adaptations can be made to fit the local healthcare system or to improve acceptability in certain clinician and patient populations, thereby increasing the likelihood of recommendation uptake and implementation [55, 56]. It is therefore somewhat expected that we would find modifications to the NICE and WHO recommendations within Australia’s guidelines. The “modifications” we identified in Australia’s guidelines may not have arisen from a formal recommendation adaptation process, however various frameworks including the ADAPTE and CAN-IMPLEMENT were designed to assist in this process [52, 55–57].

Differences in the health and cultural needs of a population can explain some of the differences in recommendations that we observed. For example, the *National guide to a preventive health assessment for Aboriginal and Torres Strait Islander people* recommends against universal screening for depression [50]. Mainstream screening tools for postnatal depression and anxiety (e.g. the Edinburgh Postnatal Depression Score) are less effective when used in Indigenous populations; the language and cultural appropriateness of these tools has been called into question [58, 59]. The importance of contextualisation is further reflected as multiple Australian guidelines recommended clinicians consider the cultural appropriateness of mental health screening tools for Indigenous, migrant and refugee populations [24, 37, 40, 45, 48, 50]. There have been multiple calls for novel approaches to perinatal mental health screening for Indigenous Australian women [24, 37, 40, 45, 48, 50, 58, 59].

Thirteen recommendations were commonly found in Australian guidelines that were not present in the NICE and WHO guidelines. Two were specific to Indigenous Australian populations, however the remaining 11 – which pertained to assessing parent-infant interactions, clinician education to promote the provision of woman-centred care, encouraging rooming in practices, and discouraging early pacifier use in breastfed infants - would be appropriate in most settings and populations internationally. We suggest that guideline producers internationally consider including these important recommendations within the scope of their own postnatal care guidelines if they do not do so already. Guideline producers and clinicians, both in Australia and internationally, should consider prioritising recommendations that work towards equity for Indigenous, migrant, and refugee populations, as these groups frequently face poor maternal and neonatal health outcomes compared to the general population [60, 61].

### Study strengths and limitations

This review had several strengths. We followed international standards for scoping review conduct and reporting, ensuring our findings were transparent and reliable. Our novel analytical approach allowed us to identify any inconsistencies in Australia’s postnatal guideline recommendations. The review was not without limitations. Our main method to find Australian guidelines was through a manual search of websites of known guideline development organisations. The choice of websites and organisations was informed by our team’s thorough knowledge of the Australian maternity care landscape. To mitigate against the possibility of missing eligible guidelines, we used an additional search of the Trip Pro database, which resulted in the replacement of one guideline [62] with a newer edition [48]). We also acknowledge that by only including recommendations related to the first 8 weeks after birth, we are perpetuating the view that postnatal care ends at this timepoint. Further research that explores long-term postnatal care recommendations, an often overlooked but essential component of care, is required [63].

## Conclusion

We identified 31 Australian postnatal care guidelines, most were moderate to high quality. Most of Australia’s guideline recommendations agree with international standards, however there are disagreements and common modifications. Furthermore, Australia lacks a single, broad postnatal care guideline for nationwide use, and this is a potential barrier to providers implementing high-quality care. Consolidating Australian recommendations, reducing disagreements and unjustified modifications, and filling recommendation gaps, will assist in the provision of high-quality, standardised postnatal care, to improve the health and wellbeing of women and newborns.

### Electronic supplementary material

Below is the link to the electronic supplementary material.

Supplementary Material 1

Supplementary Material 2

Supplementary Material 3

Supplementary Material 4

Supplementary Material 5

## Data Availability

All data generated or analysed during this study are included in this published article (and its supplementary information files).

## Abbreviations

NICE: National Institute for Health and Care Excellence
WHO: World Health Organization
PRISMA: ScR–Preferred Reporting Items for Systematic reviews and Meta–Analyses extension for scoping reviews
AGREE II: Appraisal of Guidelines for Research and Evaluation II
LEAPP: Living Evidence for Australian Pregnancy and Postnatal care Guidelines
