# Supplementary material for: How do postnatal care guidelines in Australia compare to international standards? A scoping review and comparative analysis
Source: BMC Pregnancy Childbirth. 2024 Feb 9;24:121. doi: 10.1186/s12884-024-06295-4 (PMC10854083; doi:10.1186/s12884-024-06295-4)
Supplement: Supplementary file 2 — Supplementary Material 2 [file 12884_2024_6295_MOESM2_ESM.docx]

### Supplementary file 2: List of websites searched to find Australia’s national, state, and territory postnatal care guidelines

| **Organisation** | **Type of organisation** | **Location** | **URL** |
| --- | --- | --- | --- |
| Australian Government Department of Health | Government department | Australia (National) | <https://www.health.gov.au> |
| The Royal Australian College of General Practitioners | Professional college | Australia (National) | <https://www.racgp.org.au> |
| The Royal Australian and New Zealand College of Obstetricians and Gynaecologists | Professional college | Australia and New Zealand (Bi-National) | <https://ranzcog.edu.au> |
| Australian College of Midwives | Professional college | Australia (National) | <https://www.midwives.org.au> |
| National Health and Medical Research Council | Government department | Australia (National) | <https://www.nhmrc.gov.au> |
| Australian Breastfeeding Association | Professional association | Australia (National) | <https://www.breastfeeding.asn.au> |
| Perinatal Society of Australia & New Zealand | Specialty society | Australia and New Zealand (Bi-national) | <https://psanz.com.au> |
| Society of Obstetric Medicine of Australia and New Zealand | Specialty society | Australia and New Zealand (Bi-national) | <https://www.somanz.org> |
| Maternal, Child & Family Health Nurses Australia | Professional association | Australia (National) | <https://www.mcafhna.org.au> |
| Council of Remote Area Nurses of Australia | Professional association | Australia (National) | <https://crana.org.au> |
| Australian College of Rural & Remote Medicine | Professional college | Australia (National) | <https://www.acrrm.org.au> |
| ACT Government Health | Government department | Australian Capital Territory | <https://health.act.gov.au> |
| NSW Health | Government department | New South Wales (State) | <https://www.health.nsw.gov.au> |
| NT Health | Government department | Northern Territory | <https://health.nt.gov.au> |
| Queensland Health | Government department | Queensland (State) | <https://www.health.qld.gov.au> |
| SA Health | Government department | South Australia (State) | <https://www.sahealth.sa.gov.au> |
| Tasmanian Government Department of Health | Government department | Tasmania (State) | <https://www.health.tas.gov.au> |
| Victorian Government Department of Health | Government  department | Victoria (State) | <https://www.health.vic.gov.au> |
| Government of Western Australia Department of Health | Government  department | Western Australia (State) | <https://ww2.health.wa.gov.au> |
| Centre of Perinatal Excellence | Non-profit organisation | Australia (National) | <https://www.cope.org.au> |
