# Supplementary material for: How do postnatal care guidelines in Australia compare to international standards? A scoping review and comparative analysis
Source: BMC Pregnancy Childbirth. 2024 Feb 9;24:121. doi: 10.1186/s12884-024-06295-4 (PMC10854083; doi:10.1186/s12884-024-06295-4)
Supplement: Supplementary file 3 — Supplementary Material 3 [file 12884_2024_6295_MOESM3_ESM.docx]

### Supplementary file 3. Trip database search strategy
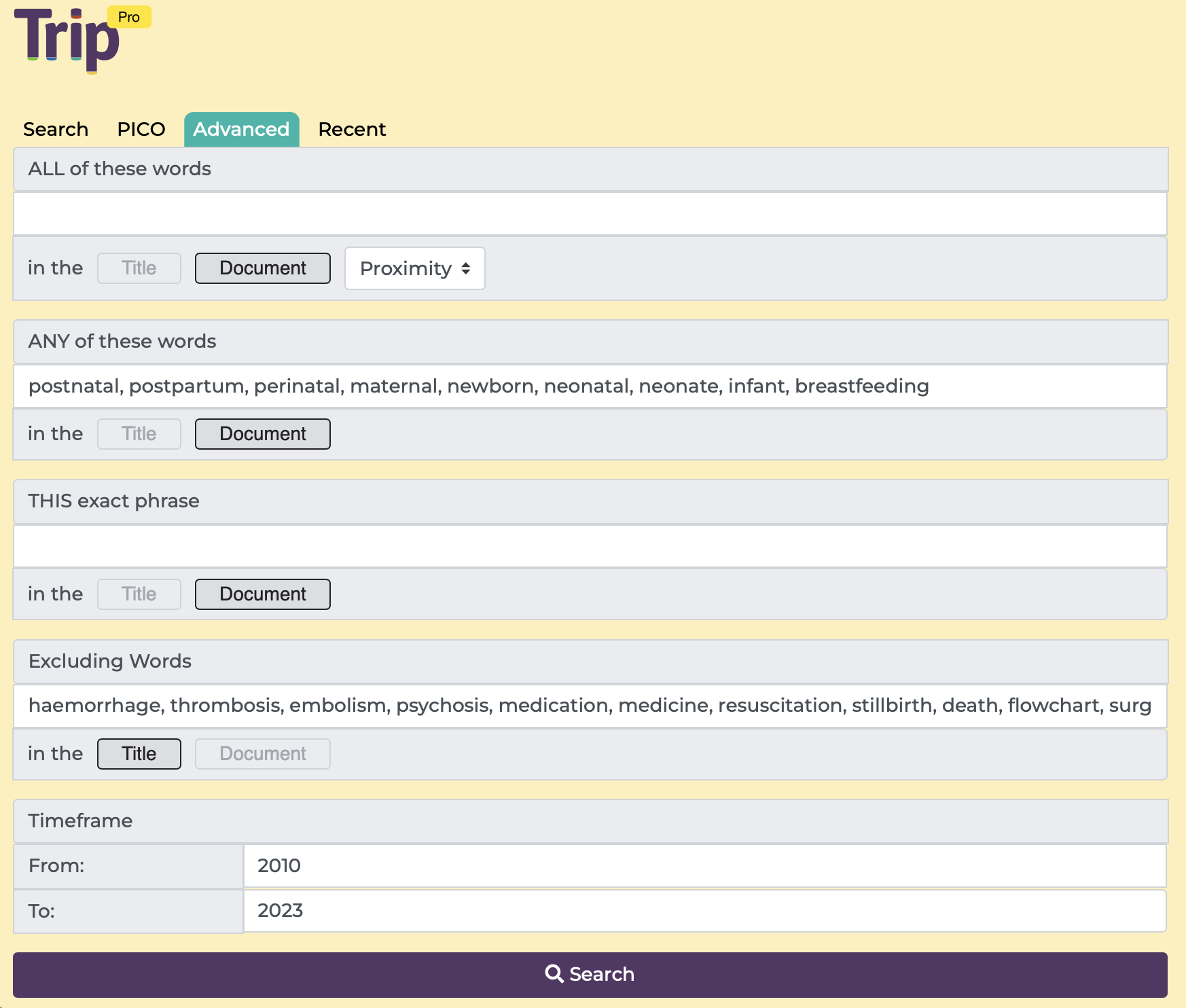

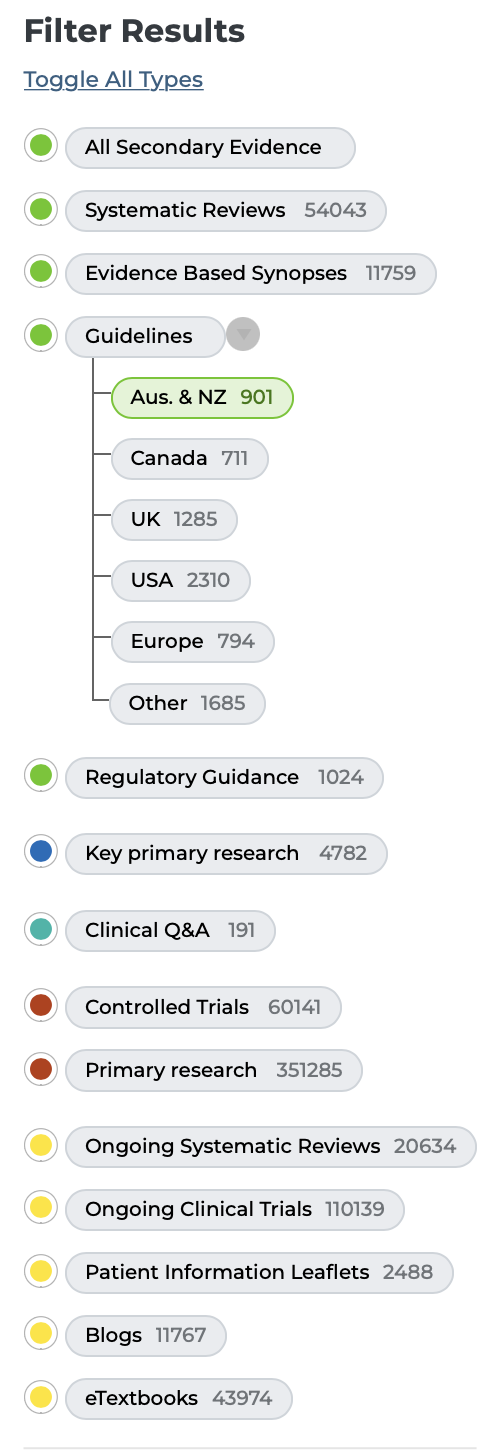


ANY of these words (in document):

# postnatal, postpartum, perinatal, maternal, newborn, neonatal, neonate, infant, breastfeeding

EXCLUDING words (in title):

# haemorrhage, thrombosis, embolism, psychosis, medication, medicine, resuscitation, stillbirth, death, flowchart, surgical, surgery

Timeframe: 2010 - 2023

Filters: Guidelines in Australia and New Zealand
