# Supplementary material for: How do postnatal care guidelines in Australia compare to international standards? A scoping review and comparative analysis
Source: BMC Pregnancy Childbirth. 2024 Feb 9;24:121. doi: 10.1186/s12884-024-06295-4 (PMC10854083; doi:10.1186/s12884-024-06295-4)
Supplement: Supplementary file 4 — Supplementary Material 4 [file 12884_2024_6295_MOESM4_ESM.docx]

## Supplementary file 4: Mapping postnatal recommendations

Key:

Any disagreements to recommendations in Australia’s guidelines

Three or more modifications to recommendations in Australia’s guidelines

Recommendations that are covered in six or fewer (<20%) of Australia’s guidelines (absent or not in scope of 25 or more guidelines)

## Recommendations: Assessment of the woman

| **Assessment of the woman recommendations** | **Number of Australian guidelines that:**   - Agree with recommendation - Partially agree with recommendation - Present a modification of recommendation - Disagree with recommendation - Recommendation is absent | **Relevant NICE guideline recommendations** | **Relevant WHO guideline recommendations** |
| --- | --- | --- | --- |
| 1.  Discuss and provide information to women regarding what to expect in the postnatal period including:   - Symptoms and signs of potential postnatal mental health problems and how to seek help - The importance of pelvic floor exercises, how to do them and when to seek help - Fatigue - Factors such as nutrition and diet, physical activity, smoking, alcohol consumption and recreational drug use - Contraception - Sexual intercourse - Safeguarding concerns, including domestic abuse | **Agree: 3 guidelines** (20,38,39) | Agree:  1.2.1  Partially agree:  1.1.13 - Provide information before discharge about the importance of pelvic floor exercises, and who to contact if any concerns arise at different stages. | Partially agree:  24 – Provide information about contraception.  47 – Provide information to prepare women and parents for discharge including the use of written/digital and pictorial educational materials. |
|  | **Partially agree: 19 guidelines**  Of these 19 guidelines:   - 8 guidelines provided information about symptoms and signs of mental health problems and how to seek help (24,36,37,40,46–48,50). - 2 guidelines provided information about the importance of pelvic floor exercises, how to do them and when to seek help (21,31). - 3 guidelines provided information about fatigue/sleep (24,45,48). - 8 guidelines provided information about factors such as nutrition and diet (22,23,28,35,42,43,47,48). - 2 guidelines provided information about factors such as physical activity (35,48). - 6 guidelines provided information about smoking (28,35,40,44,47,50). - 7 guidelines provided information about alcohol consumption (28,35,37,40,42,43,45). - 6 guidelines provided information about recreational drug use (35,37,40,43,45,46). - 5 guidelines provided information about contraception (28,30,35,42,43). - 5 guidelines provided information about sexual intercourse (21,30,31,40,46). - 10 guidelines provided information about safeguarding concerns, including domestic abuse (30,36,37,40,44–48,50). |  |  |
|  | **Modifications: 0 guidelines** |  |  |
|  | **Disagree: 0 guidelines** |  |  |
|  | **Absent from 0 guidelines** where the recommendation was in scope.  Recommendation not in scope of 9 guidelines. |  |  |
| 2.  Inform women of when to seek medical care including if they have:   - Sudden or very heavy vaginal bleeding, or persistent or increased vaginal bleeding - Abdominal, pelvic or perineal pain, fever, shivering, or vaginal discharge with an unpleasant smell - Leg swelling and tenderness, or shortness of breath - Chest pain - Persistent or severe headache, migraine, intracranial pathology or infection - Worsening reddening and swelling of breasts persisting for more than 24 hours despite self-management - Symptoms or signs of potentially serious conditions that do not respond to treatment. | **Agree: 1 guideline** (20) | Agree:  1.2.4  Partially agree:  1.2.13 – Discuss with women what vaginal bleeding to expect after birth and when to seek medical advice. | Modification:  46 – Before discharge assess the woman’s and other caregiver’s skills and confidence to care for the woman and newborn, including care-seeking behaviours. |
|  | **Partially agree: 6 guidelines**  Of these 6 guidelines:   - 2 guidelines recommended women seek support if experiencing heavy vaginal bleeding (28,39). - 1 guideline recommended women seek support if experiencing a fever (28). - 2 guidelines recommended women visualise their own perineum and seek medical review if concerned with healing or if there are signs of infection (21,31). - 2 guidelines recommended women seek medical care if they experience worsening reddening and swelling of breasts persisting for more than 24 hours despite self-management (28,35). - 1 guideline recommended discussing the various signs and symptoms with women and referring or consulting with other health providers when indicated (46). - 0 guidelines recommended women seek care for the following signs or symptoms: abdominal or pelvic pain, shivering, leg swelling and tenderness, shortness of breath, persistent or severe headache/migraine. |  |  |
|  | **Modifications: 1 guideline**   - 1 guideline recommended advising women of the risks associated with her level of perineal injury and the benefits of follow-up for ongoing symptoms (22). |  |  |
|  | **Disagree: 0 guidelines** |  |  |
|  | **Absent from 2 guidelines** where the recommendation was in scope.  Recommendation not in scope of 21 guidelines. |  |  |
| 3.  Assess psychological and emotional wellbeing using a validated instrument and refer for further assessment and management when concerned. | **Agree: 11 guidelines** (20,24,30,36,39,40,43–46,48) | Agree:  1.2.2 | Agree:  18 |
|  | **Partially agree: 2 guidelines**  Of these 2 guidelines:   - 1 guideline recommended assessing psychological and emotional wellbeing, however did not specify a validated instrument should be used to conduct the assessment (38). - 1 guideline recommended assessing emotional wellbeing but did not specify referring for follow up when concerned (32). |  |  |
|  | **Modifications: 2 guidelines**   - 1 guideline advised there is insufficient evidence to recommend for or against universal screening for postnatal depression, however GPs should be alert to signs and symptoms of depression, especially for women at high risk (47). - 1 guideline recommended using the Edinburgh Postnatal Depression Scale (EPDS) only where indicated from ‘case finding’ questions (37). |  |  |
|  | **Disagree: 1 guideline**   - 1 guideline recommended against universal screening for depression but does recommend assessing emotional wellbeing in a sub-population (women who are victims of or are at risk of family violence) (50). |  |  |
|  | **Absent from 0 guidelines** where the recommendation was in scope.  Recommendation not in scope of 15 guidelines. |  |  |
| 4.  In the first 24 hours after birth physical assessments should include:   - Regular assessments of vaginal bleeding, uterine tonus, fundal height, temperature, and heart rate - Blood pressure shortly after birth and if normal again at 6 hours | **Agree: 1 guideline** (20) | Partially agree:  1.2.3 – At each postnatal contact assess vaginal bleeding, and signs and symptoms of infection, thromboembolism, and pre-eclampsia. | Agree:  1 |
|  | **Partially agree: 2 guidelines**  Of these 2 guidelines:   - 1 guideline recommended assessing for displaced uterine fundus (23). - 1 guideline recommended assessing for hypertension, postpartum haemorrhage, pulmonary embolism, stroke, infection, and thromboembolism and referring or consulting other health providers when indicated (46). |  |  |
|  | **Modifications: 0 guidelines** |  |  |
|  | **Disagree: 0 guidelines** |  |  |
|  | **Absent from 0 guidelines** where the recommendation was in scope.  Recommendation not in scope of 28 guidelines. |  |  |
| 5.  At subsequent postnatal contacts the following should be assessed:   - General well-being - Symptoms and signs of infection, thromboembolism, anaemia, pre-eclampsia - Pain (uterine, perineal) - Vaginal discharge and bleeding - Bladder and bowel function including incontinence - Nipple and breast discomfort and inflammation symptoms - Fatigue - Backpain - Headache - Perineal healing and hygiene (vaginal births) - Wound healing and signs of infection (caesarean births) | **Agree: 2 guidelines** (20,46) | Agree:  1.2.3 | Agree:  1 |
|  | **Partially agree: 10 guidelines**  Of these 10 guidelines:   - 2 guidelines recommended assessing general well-being (30,38). - 0 guidelines recommended assessing specifically for symptoms and signs of infection, thromboembolism, anaemia, and pre-eclampsia. - 3 guidelines recommended assessing for pain (uterine, perineal) (21,31,39). - 2 guidelines recommended assessing vaginal discharge and bleeding (30,39). - 8 guidelines recommended assessing bladder and/or bowel function including incontinence (21–23,30,31,38,39,47). - 5 guidelines recommended assessing nipple and breast discomfort and inflammation symptoms (30,35,38,39,43). - 1 guideline recommended assessing for fatigue (38). - 1 guideline recommended assessing for backpain (39). - 1 guideline recommended assessing for headache (39). - 3 guidelines recommended assessing perineal healing and hygiene (21,30,31). - 1 guideline recommended assessing caesarean wound healing and signs of infection (39). |  |  |
|  | **Modifications: 0 guidelines** |  |  |
|  | **Disagree: 0 guidelines** |  |  |
|  | **Absent from 1 guideline** where the recommendation was in scope.  Recommendation not in scope of 18 guidelines. |  |  |
| 6.  If a woman seeks medical advice about vaginal bleeding after birth, assess the severity, be aware of risk factors for postpartum haemorrhage, and of the factors that can worsen the consequences of secondary postpartum haemorrhage (anaemia, weight <50kg at first pregnancy appointment). | **Agree: 1 guideline** (20) | Agree:  1.2.14 | Absent |
|  | **Partially agree: 1 guideline**   - 1 guideline recommended discussing with women the signs and symptoms of postpartum haemorrhage and secondary postpartum haemorrhage and consulting or referring with other health providers when indicated (46). |  |  |
|  | **Modifications: 2 guidelines**   - 2 guidelines recommended women with anaemia should be medically reviewed and treated (with iron therapy or dietary advice) as indicated (21,31). |  |  |
|  | **Disagree: 0 guidelines** |  |  |
|  | **Absent from 3 guidelines** where the recommendation was in scope.  Recommendation not in scope of 24 guidelines. |  |  |
| 7.  As part of assessing for perineal wound healing, women should be asked at each postnatal contact if they have any concerns about:   - Pain not resolving or worsening - Increasing need for pain relief - Discharge that has a strong or unpleasant smell - Swelling - Wound breakdown. | **Agree: 2 guidelines** (20,21) | Agree:  1.2.15 | Partially agree:  1 – At each postnatal contact after the initial 24 hours enquiries should be made regarding healing of the perineal wound and lochia. |
|  | **Partially agree: 6 guidelines**  Of these 6 guidelines:   - 2 guidelines recommended women report any concerns they have about perineal healing, or if they notice signs of wound breakdown or infection, to a midwife or GP (31,46). - 1 guideline provided a non-specific recommendation to ask women about their physical recovery after birth (38). - 1 guideline recommended considering the need for analgesia for perineal pain if a woman has not voided in four hours since previously recorded void or four hours since catheter removal (23). - 1 guideline recommended asking women about perineal pain and discomfort at the four-week maternal and child health appointment (not all contacts) (39). - 1 guideline recommended asking women about vaginal discharge/lochia in addition to visually assessing the wound (30). |  |  |
|  | **Modifications: 1 guideline**   - 1 guideline recommended that women who sustained a third or fourth degree tear attend a six-week follow-up appointment where the following should be assessed: control of bowel motions, control of flatus, faecal urgency, offensive vaginal discharge, urinary continence, pelvic floor muscle activation, ongoing perineal discomfort (22). |  |  |
|  | **Disagree: 0 guidelines** |  |  |
|  | **Absent from 1 guideline** where the recommendation was in scope.  Recommendation not in scope of 21 guidelines. |  |  |
| 8.  Consider a validated pain scale to monitor perineal pain. | **Agree: 0 guidelines** | Agree:  1.2.17 | Absent |
|  | **Partially agree: 0 guidelines** |  |  |
|  | **Modifications: 0 guidelines** |  |  |
|  | **Disagree: 0 guidelines** |  |  |
|  | **Absent from 10 guidelines** where the recommendation was in scope.  Recommendation not in scope of 21 guidelines. |  |  |
| 9.  If the woman or the healthcare professional has concerns about perineal healing or if the woman asks for reassurance, offer or arrange an examination of the perineum by a midwife or a doctor. | **Agree: 4 guidelines** (21,31,39,46) | Agree:  1.2.18 | Absent |
|  | **Partially agree: 3 guidelines**   - All 3 of these guidelines recommended that any physical concerns a woman has should warrant an examination of vital signs and a referral for medical review where indicated. They do not specifically mention concerns about perineal healing (20,30,38). |  |  |
|  | **Modifications: 1 guideline**   - 1 guideline recommended that women with a third- or fourth-degree tear who report concerns or symptoms of incontinence or pain at their six week follow up appointment should be referred to a colorectal specialist (22). |  |  |
|  | **Disagree: 0 guidelines** |  |  |
|  | **Absent from 2 guidelines** where the recommendation was in scope.  Recommendation not in scope of 21 guidelines. |  |  |
| 10.  At 6 to 8 weeks after the birth, a GP should:   - Carry out an assessment including the points in recommendations 1-3 and 5, taking into account the time since the birth. - Respond to any concerns, which may include referral to specialist services in either secondary care or other healthcare services such as physiotherapy. | **Agree: 4 guidelines** (21,22,30,31) | Agree:  1.2.7 | Agree:  Combining recommendations 1, 18, and 44 |
|  | **Partially agree: 6 guidelines**  Of these 6 guidelines:   - 4 guidelines recommended assessing emotional wellbeing plus or minus the use of the EPDS at either 4 weeks (37), 6 weeks (40), or 6-12 weeks (24,48) postpartum. - 1 guideline recommended routine follow up with a GP (no time or types of assessments specified) and with a qualified health professional for a breastfeeding evaluation (no time specified) (35). - 1 guideline specific for GPs recommended being alert for signs and symptoms of mental illness especially in high-risk women and assessing for urinary incontinence. No time for assessment specified (47). |  |  |
|  | **Modifications: 6 guidelines**   - 4 guidelines recommended the assessment be carried out by a provider other than a GP, for example by a maternal and child health nurse (38,39,44) or an obstetrician (45). - 2 guidelines recommended alternate timing or frequency of assessments, for example, at two and six weeks (20), or no follow up (specific to assessing bladder function when normal postpartum void parameters are met) (23). |  |  |
|  | **Disagree: 0 guidelines** |  |  |
|  | **Absent from 0 guidelines** where the recommendation was in scope.  Recommendation not in scope of 15 guidelines. |  |  |
| **Additional recommendations about assessment of the woman found in four or more of Australia’s guidelines** | | | |
| A1.  Be aware of common risk factors for poor mental health outcomes and conduct comprehensive psychosocial assessments of postpartum women and families to identify those at higher risk of poor mental health outcomes. Validated tools to measure psychosocial risk such as the Postnatal Risk Questionnaire (PNQR) are best used in conjunction with a tool that screens for current symptoms of depression and anxiety (i.e. the Edinburgh Postnatal Depression Score). | **Agree: 6 guidelines** (36,40,44,46–48) | Partially agree:  1.2.1 – Discuss with women the signs and symptoms of potential postnatal mental health problems, and safeguarding concerns including domestic abuse. | Partially agree:  46 – Prior to discharge from health facility assess the woman’s emotional wellbeing and the home environment. |
| A2.  Health workers with appropriate training are encouraged to assess parent-infant interactions using validated tools such as The Mother Object Relations Scale, the Mother-to-Infant Bonding Scale, or the Postpartum Bonding Questionnaire. Health workers without training are also encouraged to observe parent-infant interactions and refer on when concerned. | **Agree: 5 guidelines** (30,37,40,44,48) | Absent | Partially agree:  46 – Prior to discharge from the health facility assess the skills and confidence of the parents and caregivers to care for the newborn. |
| A3.  Reporting requirements and procedures for child protection services should be clearly communicated to health providers working in facilities providing postpartum care to women and families. | **Agree: 4 guidelines** (37,44,48,50) | Partially agree:  1.1.8 – Ensure effective and prompt communication between health providers about any safeguarding issues, or concerns about the baby’s health and care. | Absent |
| A4.  The language and cultural appropriateness of mental health screening tools and management interventions should be considered for Indigenous Australian populations and migrant and refugee populations. | **Agree: 6 guidelines**   - 6 guidelines recommended this care practice for Indigenous Australian populations (24,37,40,45,48,50). - 5 guidelines recommended this care practice migrant and refugee populations (24,37,40,45,48). | Absent | Absent |
| A5.  Provide Indigenous Australian women the option to have an Aboriginal health worker present at postnatal appointments, particularly for appointments of a sensitive nature (e.g. mental health screening and management). | **Agree: 4 guidelines** (25,37,48,50) | Absent | Absent |
| A6.  Health workers should be aware of the local referral pathways available to postpartum women and families identified as having significant mental health issues and those at high risk of self-harm or suicide. | **Agree: 9 guidelines** (20,24,37,40,44–46,48,50) | Partially agree:  1.2.2 – At each postnatal contact assess the woman’s psychological wellbeing. If there are concerns, arrange for further assessment and follow-up. | Partially agree:  19 – Psychosocial and/or psychological interventions are recommended to prevent postpartum depression and anxiety. |
| A7.  Postnatal care should adhere to the principles of woman centred care. Clinicians should ensure they communicate with women clearly and in a supportive manner, they encourage women to be involved in their own care decisions, and they respect women’s care choices. | **Agree: 6 guidelines** (20,32,37,40,45,48) | Agree but not stated explicitly in a recommendation:  The principles section of the NICE guideline considers woman- and family-centred care practices. | Agree but not stated explicitly in a recommendation:  The overarching aim of the WHO guidelines is to promote respectful, individualised and person-centred care. |
| A8.  Health workers should have sufficient and ongoing training that allows them to competently communicate with, assess, and manage postnatal women, newborns, and families. Training should include information about:   - Best practice in specific assessment procedures (including perineal assessments, mental health screening, newborn hearing screening and hip assessments). - Techniques to communicate with women that promote a woman-centred approach to care interactions. | **Agree: 8 guidelines**   - 8 guidelines recommended sufficient and ongoing training about specific procedures and assessments (20,27,31,32,37,40,45,48). - 3 guidelines recommended sufficient and ongoing training about communication methods needed for a woman-centred approach to care (37,45,48). | Partially agree:  1.5.13 – Health providers with skills and competencies in breastfeeding support should assess breastfeeding to identify and address any concerns. | Partially agree:  43b – Health providers who provide infant feeding services should have sufficient knowledge, competence, and skills to support women to breastfeed. |

## Recommendations: Assessment of the newborn

| **Assessment of the newborn recommendations** | **Number of Australian guidelines that:**   - Agree with recommendation - Partially agree with recommendation - Present a modification of recommendation - Disagree with recommendation - Recommendation is absent | **Relevant NICE guideline recommendations** | **Relevant WHO guideline recommendations** |
| --- | --- | --- | --- |
| 11.  At each postnatal contact, ask parents if they have any concerns about their baby's general wellbeing, feeding or development. Review the history and assess the baby's health, including physical inspection and observation. If there are any concerns (from parents or from clinical assessment findings), take appropriate further action. | **Agree: 7 guidelines** (20,30,32,35,38,39,44) | Agree:  1.3.1 | Modified:  25 – Recommendation is mostly present, however instead of asking parents if they have concerns, WHO recommendation 25 encourages parents to seek care when they themselves identify concerns. |
|  | **Partially agree: 11 guidelines**  Of these 11 guidelines:   - 3 guidelines recommended physical observation of the newborn (28,37,48). - 1 guideline recommends observing the newborn and their behaviour, and documenting feeding and output (43). - 1 guideline recommended regular checks of child health and wellbeing and family functioning in the first year of life (40). - 1 guideline recommended reviewing history and physical observation of the newborn in relation to performing a hearing assessment (25). - 2 guidelines recommended reviewing history, assessing risk of jaundice, and taking appropriate action where indicated (26,33). - 1 guideline recommended midwives take appropriate action (discuss with parents, consult or refer to other health providers) when a newborn observation or assessment has an abnormal finding (46). - 1 guideline recommended enquiring about developmental progress including behaviours that indicate normal hearing and vision (47). - 1 guideline recommended documenting carer concerns and any barriers they perceive to breastfeeding (50). |  |  |
|  | **Modifications: 0 guidelines** |  |  |
|  | **Disagree: 0 guidelines** |  |  |
|  | **Absent from 1 guideline** where the recommendation was in scope.  Recommendation not in scope of 12 guidelines. |  |  |
| 12.  At each postnatal contact recognise and assess for the following ‘red flags’ for serious illness in young babies:   - Appearing ill to a healthcare professional - Appearing pale, ashen, mottled or blue (cyanosis) - Unresponsive, unrousable - Having a weak, abnormally high-pitched or continuous cry - Non-blanching rash - Bulging fontanelle - Neck stiffness - Focal neurological signs - Diarrhoea associated with dehydration - Frequent forceful (projectile) vomiting - Bilious vomiting (green or yellow-green vomit) - Abnormal breathing pattern, such as:   － Grunting respirations  － Increased respiratory rate  (over 60 breaths/minute)  － Chest indrawing   - Temperature of 38°C or over or under 36°C - Seizures / history of convulsions - Yellow palms and soles at any age - Not feeding well/refusing feeds. | **Agree: 1 guideline (32)** | Partially agree:  1.3.1, 1.4.7, and 1.4.9 – All of care practice present when the above recommendations are combined, except for recognising yellow palms and soles at any age as a ‘red’ flag’. | Partially agree and modified:  25 – Recognises the ‘red flags’ listed in the recommendation from increased respiratory rate onwards. Modification is due to different temperature limits for fever (>37.5°C) and low body temperature (<35.5°C). |
|  | **Partially agree: 9 guidelines**  Of these 9 guidelines, the following guidelines recommended recognising each of the ‘red flags’ as signs of serious illness in young babies:   - 6 guidelines recognised when a young baby appears ill to a health provider (26,28,35,37,39,46). - 1 guideline recommended recognising when a young baby appears ashen, mottled or blue (46). - 4 guidelines recommended recognising when a young baby is unresponsive or unrousable (26,30,35,39). - 2 guidelines recommended recognising a weak, abnormally high-pitched or continuous cry (35,39). - 0 guidelines recommended recognising a non-blanching rash. - 0 guidelines recommended recognising a bulging fontanelle, while 1 guideline recognised a depressed fontanelle (35). - 0 guidelines recommended recognising neck stiffness. - 4 guidelines recommended recognising focal neurological signs, primarily loss of muscle tone (28,30,35,39). - 0 guidelines recommended recognising diarrhoea associated with dehydration. - 3 guidelines recommended recognising frequent forceful vomiting (30,33,46). - 1 guidelines recommended recognising bilious vomiting (46). - 3 guidelines recommended recognising abnormal breathing patterns (20,30,39). - 4 guidelines recommended recognising high and or low temperatures (20,28,35,46). - 2 guidelines recommended recognising seizures / history of convulsions (26,46). - 3 guidelines recommended recognising yellow palms and soles at any age (jaundice) (26,33,46). - 2 guidelines recommended recognising skin colour but was not specific about what they were noting (35,39). - 7 guidelines recommended recognising not feeding well/refusing feeds (26,28,30,33,35,39,46). |  |  |
|  | **Modifications: 3 guidelines**  Of these 3 guidelines:   - 1 guideline recommended informing parents about how to recognise signs of serious illness in their infant (38). - 1 guideline recommended clinicians be aware of the signs and symptoms of an alternate ‘red flag’, insufficient milk. Signs include infrequent stools, scant/infrequent urination, and failure to gain weight/excessive weight loss (43). This recommendation was also present in 2 of the partially agreeing guidelines (35,46). - 1 guideline recommended maintaining a high level of suspicion for infants with the following risk factors: possible fetal alcohol syndrome, microcephaly, convulsions, prematurity (50). |  |  |
|  | **Disagree: 0 guidelines** |  |  |
|  | **Absent from 1 guideline** where the recommendation was in scope.  Recommendation not in scope of 17 guidelines. |  |  |
| 13.  Be aware of the possible significance of a change in the baby's behaviour or symptoms, such as refusing feeds or a change in the level of responsiveness. | **Agree: 7 guidelines** (20,26,28,30,33,35,39) | Agree:  1.4.7 | Partially agree:  25 – Newborns who are not feeding well or who have no spontaneous movement require further evaluation. Does not specify the significance of a change in behaviour/feeding. |
|  | **Partially agree: 3 guidelines**  Of these 3 guidelines:   - 1 guideline recommended being aware of and taking further action if an infant becomes difficult to settle or irritable most of the time, or if they experience serious and continued feeding problems (37). - 1 guideline recommended being aware of the significance of a change in alertness and state of consciousness (32). - 1 guideline recommended midwives consult with or refer to other health providers after an abnormal newborn examination finding (46). |  |  |
|  | **Modifications: 0 guidelines** |  |  |
|  | **Disagree: 0 guidelines** |  |  |
|  | **Absent from 2 guidelines** where the recommendation was in scope.  Recommendation not in scope of 19 guidelines. |  |  |
| 14.  If a baby is thought to be seriously unwell based on a 'red flag' (see recommendation 12) or on an overall assessment of their condition, clinicians should arrange an immediate assessment with an appropriate emergency service. If the baby's condition is immediately life-threatening, dial 000. | **Agree: 5 guidelines** (20,32,33,38,46) | Agree:  1.4.10 | Partially agree:  25 – If one or more ‘red flags’ are present newborn should be referred for further evaluation. No mention of emergency services. |
|  | **Partially agree: 3 guidelines**  Of these 3 guidelines:   - All 3 guidelines recommended escalating the situation by referring to and having an assessment performed by a more senior or specialised clinician (28,35,43). - 0 guidelines recommended communicating with emergency services or calling 000. |  |  |
|  | **Modifications: 1 guideline**   - This guideline about neonatal jaundice recommended multiple care pathways for newborns with varying levels of risk/symptoms of jaundice. Newborns with more severe symptoms are recommended to be reviewed by paediatricians or neonatologists and transferred to level 6 health facilities (with neonatal intensive unit capabilities) (26). |  |  |
|  | **Disagree: 0 guidelines** |  |  |
|  | **Absent from 3 guidelines** where the recommendation was in scope.  Recommendation not in scope of 19 guidelines. |  |  |
| 15.  Parents and family should be encouraged to seek health care early if they identify any of the ‘red flags’ (see recommendation 20) between postnatal care visits. | **Agree: 5 guidelines** (20,28,32,33,38) | Modified:  1.4.1 – Listen carefully to parents’ concerns about their baby’s health and treat their concerns as a possible indicator of serious illness. | Agree:  25 |
|  | **Partially agree: 1 guideline**   - This 1 guideline recommended advising parents that they should seek medical review for any visible jaundice after the age of two weeks. No other ‘red flags’ mentioned (26). |  |  |
|  | **Modifications: 0 guidelines** |  |  |
|  | **Disagree: 0 guidelines** |  |  |
|  | **Absent from 4 guidelines** where the recommendation was in scope.  Recommendation not in scope of 21 guidelines. |  |  |
| 16.  Be aware that if the baby has not passed meconium within 24 hours of birth, this may indicate a serious disorder and requires medical advice. | **Agree: 3 guidelines** (32,35,46) | Agree:  1.3.2 | Absent |
|  | **Partially agree: 1 guideline**   - This 1 guideline recommended assessing stool frequency, colour and consistency but did not mention the significance of no stools within 24 hours of birth (20). |  |  |
|  | **Modifications: 3 guidelines**  Of these 3 guidelines:   - 2 guidelines recommended the number of bowel motions per day should be assessed on the fourth or fifth day of life (33,49). - 1 guideline advised that inadequate bowel motions may be an indication of inadequate intake/breastfeeding (49). - 1 guideline recommended acholic stools at any stage requires urgent medical review (26). |  |  |
|  | **Disagree: 0 guidelines** |  |  |
|  | **Absent from 0 guidelines** where the recommendation was in scope.  Recommendation not in scope of 24 guidelines. |  |  |
| 17.  Follow the recommendations on neonatal infection including how to assess for and manage the risk of early-onset neonatal infection (within 72 hours of the birth) and possible late-onset neonatal infection (more than 72 hours after the birth). See the NICE guideline on neonatal infection for more detail. | **Agree: 1 guideline** (20) | Agree:  1.4.3 | Partially agree:  25 – The ‘red flags’ (listed for recommendation 12) that require assessment at each postnatal contact can all be signs of neonatal infection. Does not specify how to manage risks of infection. |
|  | **Partially agree: 4 guidelines**  Of these 4 guidelines:   - 1 guideline recommended midwives consult with other health providers when a newborn has signs of infection to the umbilical stump, or to refer to other health providers for temperature instability (46). - 2 guidelines provide advice about the signs of infection in relation to jaundice (26,33). - 1 guideline recommended assessing temperature, alertness, and feeding but does not state these as possible signs of infection (39). |  |  |
|  | **Modifications: 0 guidelines** |  |  |
|  | **Disagree: 0 guidelines** |  |  |
|  | **Absent from 4 guidelines** where the recommendation was in scope.  Recommendation not in scope of 22 guidelines. |  |  |
| 18.  Be aware that fever may not be present in young babies with a serious infection. | **Agree: 0 guidelines** | Agree:  1.4.4 | Absent |
|  | **Partially agree: 0 guidelines** |  |  |
|  | **Modifications: 0 guidelines** |  |  |
|  | **Disagree: 0 guidelines** |  |  |
|  | **Absent from 9 guidelines** where the recommendation was in scope.  Recommendation not in scope of 22 guidelines. |  |  |
| 19.  Carry out a complete examination of the baby within 72 hours of the birth and at 6 to 8 weeks after the birth. This should include checking the baby's:   - Appearance, including colour, breathing, behaviour, activity and posture - Head (including fontanelles), face, nose, mouth (including palate), ears, neck and general symmetry of head and facial features - Eyes: opacities, red reflex and colour of sclera - Neck and clavicles, limbs, hands, feet and digits; assess proportions and symmetry - Heart: position, heart rate, rhythm and sounds, murmurs and femoral pulse volume - Lungs: respiratory effort, rate and lung sounds - Abdomen: assess shape and palpate to identify any organomegaly; check condition of umbilical cord - Genitalia and anus: completeness and patency and undescended testes in boys - Spine: inspect and palpate bony structures and check integrity of the skin - Skin: colour and texture as well as any birthmarks or rashes - Central nervous system: tone, behaviour, movements and posture; check newborn reflexes only if concerned - Hips: symmetry of the limbs, Barlow and Ortolani's manoeuvres - Cry: assess sound. | **Agree: 4 guidelines** (20,30,32,39) | Agree:  1.3.3 | Partially agree:  25 – At each postnatal contact newborns should be assessed for ‘red flags’ as listed under recommendation 12 (from increased respiratory rate onwards).  44 – Four postnatal contacts are recommended within the first 24 hours, between 48-72 hours, between 7-14 days, and during week 6 after birth. |
|  | **Partially agree: 9 guidelines**  Of these 9 guidelines:   - 1 guideline recommended carrying out an examination of the baby within 72 hours of birth (26). - 1 guideline recommended a ‘head to toe’ assessment should be performed in the woman’s room if possible but should be delayed until after the first breastfeed (35). - 1 guideline recommended midwives consult with other health providers when they encounter an abnormal finding on a newborn examination between 1 hour and 28 days after birth (46). - 1 guidelines recommended carrying out an examination of the baby at 6-8 weeks after the birth, although this guideline was specifically for babies requiring a hip ultrasound for suspected developmental hip dysplasia (27). - 1 guideline recommended assessing appearance (46). - 3 guidelines recommended assessing the head (38,44,47). - 4 guidelines recommended assessing the eyes (38,44,47,50). - 2 guidelines recommended assessing the neck and clavicles, limbs, hands, feet and digits (44,46). - 1 guideline recommended assessing the heart (47). - 0 guidelines recommended assessing the lungs. - 1 guideline recommended assessing the abdomen (47). - 1 guideline recommended assessing the genitalia and anus (47). - 0 guidelines recommended assessing the spine. - 4 guidelines recommended assessing the skin (26,44,46,47). - 3 guidelines recommended assessing the central nervous system (33,46,47), although one of these guidelines recommended always testing reflexes, not just when concerned (47). - 3 guidelines recommended assessing the hips (27,38,47). - 1 guideline recommended assessing the sound of the cry (33). |  |  |
|  | **Modifications: 0 guidelines** |  |  |
|  | **Disagree: 0 guidelines** |  |  |
|  | **Absent from 0 guidelines** where the recommendation was in scope.  Recommendation not in scope of 18 guidelines. |  |  |
| 20.  Be aware that the presence or absence of individual symptoms or signs may be of limited value in identifying or ruling out serious illness in a young baby. | **Agree: 2 guidelines** (27,32) | Agree:  1.4.8 | Absent |
|  | **Partially agree: 1 guideline**   - This 1 guideline recommends that the need for further jaundice examinations and treatment will be dependent on if the baby is otherwise thriving and feeding well, or if they are unwell/not thriving (33). |  |  |
|  | **Modifications: 2 guidelines**  Of these 2 guidelines:   - 1 guideline advised that weight gain or loss is only one aspect of wellbeing and that each newborn should be assessed on an individual basis (35). - 1 guideline recommended that a midwives decision to refer to or consult with another health provider will be dependent on the severity and the number of clinical indicators found during newborn examination (46). |  |  |
|  | **Disagree: 0 guidelines** |  |  |
|  | **Absent from 6 guidelines** where the recommendation was in scope.  Recommendation not in scope of 20 guidelines. |  |  |
| 21.  At 6 to 8 weeks, assess the baby's social smiling and visual fixing and following. | **Agree: 2 guidelines** (39,47) | Agree:  1.3.4 | Absent  Except for specifying a postnatal contact should occur at 6 weeks after birth in recommendation 44. |
|  | **Partially agree: 4 guidelines**  Of these 4 guidelines:   - 2 guidelines recommended assessing social smiling and visual fixing but did not include the timing of assessment (38,44). - 1 guideline recommended assessing visual fixing and following at 6 weeks (30). - 1 guideline recommended assessing social smiling at 6 weeks (50). |  |  |
|  | **Modifications: 0 guidelines** |  |  |
|  | **Disagree: 0 guidelines** |  |  |
|  | **Absent from 2 guidelines** where the recommendation was in scope.  Recommendation not in scope of 23 guidelines. |  |  |
| 22.  Measure weight and head circumference of babies in the first week and around 8 weeks, and at other times only if there are concerns. Plot the results on the growth chart. | **Agree: 4 guidelines** (20,30,32,38) | Agree:  1.3.5 | Absent |
|  | **Partially agree: 4 guidelines**  Of these 4 guidelines:   - 1 guideline recommended measuring both weight and head circumference but did not specify the timing of measurement (42). - 2 guidelines recommended measuring weight but did not specify the timing of measurement (26,35). - 1 guideline recommended measuring weight around 72 hours after birth, with follow up measurements to occur if weight loss of >10% of birth weight (28). |  |  |
|  | **Modifications: 4 guidelines**  Of these 4 guidelines:   - 1 guideline recommended weight gain be assessed on a four-week average but did not specify when measurements should routinely occur, however did specify that weight should be assessed in infants who are overly fussy/excessively crying (43). - 3 guidelines recommended alternate frequencies for undertaking growth assessments including at:   - At each maternal and child health appointment (39)   - Weeks 1 and 6, and months 4, 6 , 12 , and 18 (50)   - Midwifery contacts from days 0-28 (46) |  |  |
|  | **Disagree: 2 guidelines**   - These 2 guidelines recommended growth assessments be performed at each maternal and child health appointment (not just at one and eight weeks and when concerned). Both guidelines noted that parents and carers value their child being weighed and reviewing completed growth charts, and may be an incentive to attend follow up appointments (44,47). |  |  |
|  | **Absent from 0 guidelines** where the recommendation was in scope.  Recommendation not in scope of 17 guidelines. |  |  |
| 23.  If there are concerns about the baby's growth, follow the recommendations for faltering growth. This includes for babies who have lost >10% loss of birth weight in the early days or have not returned to birth weight by 3 weeks:   - Perform a clinical assessment looking for signs of dehydration, or evidence of an illness or disorder that might account for weight loss. - Assess feeding history and consider observing a feed (if appropriately skilled to do so). - Provide feeding support where appropriate. - Refer to paediatric services of there is evidence of illness, significant weight loss, or failure to respond to feeding support. | **Agree: 2 guidelines** (42,50) | Agree:  1.4.6 (in addition to the NICE guideline on faltering growth). | Absent |
|  | **Partially agree: 11 guidelines**  Of these 11 guidelines:   - 1 guideline recommended measuring weight, repeating measurements if concerned, and attaining medical input when abnormalities detected (20). - 2 guidelines recommended repeated weight measurements as needed and providing lactation/feeding support where indicated for babies at high risk/symptoms of jaundice (26,33). - 2 guidelines provided the indications for further investigations including: <10^th^ percentile, >90^th^ percentile, more than 10% loss of birth weight (32,43). - 1 guideline recommended using a 10% weight loss as an indicator for further evaluation but noted this should not be used in isolation as an indicator for supplementation (49). - 2 guidelines recommended providing families with appropriate referrals to support the child’s growth and development (38,39). - 1 guideline recommended midwives discuss with women and/or consult with other health providers if they identify issues with a newborns weight or if there are feeding problems (46). - 2 guidelines recommended measuring the newborns growth and reviewing growth charts, but did not include what to do if growth faltering detected (44,47). |  |  |
|  | **Modifications: 0 guidelines** |  |  |
|  | **Disagree: 0 guidelines** |  |  |
|  | **Absent from 1 guideline** where the recommendation was in scope.  Recommendation not in scope of 17 guidelines. |  |  |
| 24.  Carry out newborn blood spot screening in line with the national newborn blood spot screening programme. | **Agree: 4 guidelines** (20,32,39,47) | Agree:  1.3.8 | Absent |
|  | **Partially agree: 0 guidelines** |  |  |
|  | **Modifications: 0 guidelines** |  |  |
|  | **Disagree: 0 guidelines** |  |  |
|  | **Absent from 1 guideline** where the recommendation was in scope.  Recommendation not in scope of 26 guidelines. |  |  |
| 25.  Universal newborn hearing screening (UNHS) with otoacoustic emissions (OAE) or automated auditory brainstem response (AABR) is recommended for early identification of permanent bilateral hearing loss. UNHS should be accompanied by diagnostic and management services for children identified with hearing loss. | **Agree: 8 guidelines** (20,25,32,34,41,44,47,50) | Agree:  1.3.9 | Agree:  27 |
|  | **Partially agree: 3 guidelines**  Of these 3 guidelines:   - 2 guidelines recommended hearing screening with otoacoustic emissions or automated auditory brainstem response (26,39). - 0 guidelines recommended hearing screening should be accompanied by diagnostic and management services for children identified with hearing loss. - 1 guidelines recommended maternal and child health nurses utilise validated tools to assess the newborns health, including a hearing risk factor assessment (does not specify OAE or AABR) (38). |  |  |
|  | **Modifications: 2 guidelines**   - 1 guideline recommended that newborns with jaundice undergo their hearing screening after completion of phototherapy (33). - 1 guideline recommended checking for parental concerns about the baby’s hearing (30). |  |  |
|  | **Disagree: 0 guidelines** |  |  |
|  | **Absent from 1 guideline** where the recommendation was in scope.  Recommendation not in scope of 17 guidelines. |  |  |
| 26.  Universal newborn screening for abnormalities of the eye is recommended and should be accompanied by diagnostic and management services for children identified with an abnormality. Screening should involve an external examination of the eye and red reflex test, ideally performed prior to discharge from the birthing facility or at the first postnatal contact after a home birth. | **Agree: 5 guidelines** (20,32,39,44,50) | Absent | Agree:  26 |
|  | **Partially agree: 2 guidelines**  Of these 2 guidelines:   - 2 guideline recommends performing newborn screening for abnormalities of the eye (30,47). - 0 guidelines recommended eye screening should be accompanied by diagnostic and management services for children identified with an abnormality. |  |  |
|  | **Modifications: 0 guidelines** |  |  |
|  | **Disagree: 0 guidelines** |  |  |
|  | **Absent from 2 guidelines** where the recommendation was in scope.  Recommendation not in scope of 22 guidelines. |  |  |
| 27.  Universal screening for neonatal hyperbilirubinaemia by transcutaneous bilirubinometer (TcB) is recommended at health facility discharge. | **Agree: 0 guidelines** | Absent | Agree:  28 |
|  | **Partially agree: 0 guidelines** |  |  |
|  | **Modifications: 2 guidelines**   - These 2 guidelines recommended all babies should be clinically assessed daily for jaundice in hospital and during home visits by either TcB or by blanching the skin in bright natural light or white fluorescent light (20,26). |  |  |
|  | **Disagree: 2 guidelines**  These 2 guidelines did not recommend universal screening with TcB:   - 1 guideline recommended considering a TcB measurement in infants discharged at <72 hours where early follow up is unlikely to occur. This guideline also suggested TcB screening is likely justified in babies with significant risk factors (33). - 1 guideline recommended considering TcB measurement prior to discharge, following local protocols (32) |  |  |
|  | **Absent from 1 guideline** where the recommendation was in scope.  Recommendation not in scope of 26 guidelines. |  |  |
| 28.  There is insufficient evidence to recommend for or against universal screening by total serum bilirubin (TSB) at health facility discharge. | **Agree: 3 guidelines**  As this care practice states there is insufficient evidence to recommend for or against TSB screening, Australian guidelines that recommended for or against universal screening were considered to be in agreement.  3 guidelines provided specific indications for TSB use, thereby recommending against universal screening. Indications for TSB screening included:   - Infants with any visible jaundice in the first 24 hours even if only on the face or above the nipple line (26,33) - Infants born at less than 35 weeks gestation who are visibly jaundiced (33) - Infants with suspected severe jaundice or with significant risk factors (use TcB and/or TSB) (33) - Dark skinned infants with any visible jaundice at the time of the newborn screening test (TSB or plasma BR) (26) - Infants requiring close monitoring of total bilirubin levels (20). | Absent | Agree:  29 |
|  | **Partially agree: 0 guidelines** |  |  |
|  | **Modifications: 0 guidelines** |  |  |
|  | **Disagree: 0 guidelines** |  |  |
|  | **Absent from 2 guidelines** where the recommendation was in scope.  Recommendation not in scope of 26 guidelines. |  |  |
| 29.  Listen carefully to parents' concerns about their baby's health and treat their concerns as an important indicator of possible serious illness in their baby. | **Agree: 6 guidelines** (20,30,34,38,39,44) | Agree:  1.4.1 | Partially agree:  25 – Parents should be encouraged to seek health care early if they identify and ‘red flags’ in their newborn between care visits. |
|  | **Partially agree: 4 guidelines**  Of these 4 guidelines:   - 1 guideline recommended asking parents if they have any concerns but do not specify they should be treated as an important indicator of possible serious illness (47). - 3 guidelines recommended listening to parental concerns on specific subjects including routine newborn assessments and procedures (32,41), and newborns receiving phototherapy (33). |  |  |
|  | **Modifications: 1 guideline**   - This 1 guideline recommended advising women of when to seek additional support (newborn not waking for feeds, reduced/infrequent urine and stool output) as these may be signs of illness (28). |  |  |
|  | **Disagree: 0 guidelines** |  |  |
|  | **Absent from 3 guidelines** where the recommendation was in scope.  Recommendation not in scope of 17 guidelines. |  |  |
| 30.  Healthcare professionals should consider using the Baby Check scoring system:   - To supplement the clinical assessment of babies for possible illness, particularly as part of a remote assessment and - As a communication aid in conversations with parents to help them describe the baby's condition. | **Agree: 0 guidelines** | Agree:  1.4.2 | Absent |
|  | **Partially agree: 0 guidelines** |  |  |
|  | **Modifications: 4 guidelines**  All 4 of these guidelines recommended using alternate tools to elicit parental concerns about an infant’s health and/or development including the:   - Parents’ Evaluation of Developmental Status (PEDS) (38,39,47) - Ages and Stages Questionnaire (47) - Parent report questionnaires and questions in the patient-held record (50) |  |  |
|  | **Disagree: 0 guidelines** |  |  |
|  | **Absent from 7 guidelines** where the recommendation was in scope.  Recommendation not in scope of 20 guidelines. |  |  |

## Recommendations: Infant feeding

| **Infant feeding recommendations** | **Number of Australian guidelines that:**   - Agree with recommendation - Partially agree with recommendation - Present a modification of recommendation - Disagree with recommendation - Recommendation is absent | **Relevant NICE guideline recommendations** | **Relevant WHO guideline recommendations** |
| --- | --- | --- | --- |
| 31.  All babies should be exclusively breastfed from birth until 6 months of age. Women should be counselled and provided with support for exclusive breastfeeding at each postnatal contact. | **Agree: 5 guidelines** (28,35,42,43,49) | Modified:  1.5.2 – Provide breastfeeding support and information including how it can have benefits even if only done for a short time. | Agree:  42 |
|  | **Partially agree: 4 guidelines**  Of these 4 guidelines:   - 0 guidelines recommended babies should be exclusively breastfed from birth until 6 months of age. - 4 guidelines recommended women should be counselled and provided with support for exclusive breastfeeding at each postnatal contact (20,30,38,39). |  |  |
|  | **Modifications: 1 guideline**   - This 1 guideline recommended several, somewhat conflicting, exclusive breastfeeding durations (50):   - For infants born without risk factors for iron deficiency anaemia, exclusive breastfeeding is recommended until 6 months of age.   - For infants born with low birth weight (<2500 grams), prematurity (<37 weeks), or to women with maternal anaemia, exclusive breastfeeding is recommended until 4 months of age.   - For all infants, introduce iron-rich foods at around 4-6 months.   - To reduce the risk of infants acquiring acute otitis media, promote exclusive breastfeeding for at least 3 months. |  |  |
|  | **Disagree: 1 guideline**   - This 1 guideline recommended encouraging and supporting exclusive breastfeeding until 4-6 months of age, with breastfeeding recommended to continue until 12 months of age and thereafter as long as is mutually desired (47). |  |  |
|  | **Absent from 2 guidelines** where the recommendation was in scope.  Recommendation not in scope of 18 guidelines. |  |  |
| 32.  Facilities providing maternity and newborn services should have a clearly written breastfeeding policy that is routinely communicated to staff and parents. | **Agree: 5 guidelines** (28,35,42,43,49) | Absent | Agree:  43a |
|  | **Partially agree: 1 guideline**   - This 1 guideline recommended having monitoring criteria to assess breastfeeding in women attending maternal and child health services but did not include information on a comprehensive breastfeeding policy (38). |  |  |
|  | **Modifications: 1 guideline**   - This 1 guideline recommended having a formal policy to guide staff in the management of infection prevention and control of expressed breastmilk (29). |  |  |
|  | **Disagree: 0 guidelines** |  |  |
|  | **Absent from 4 guidelines** where the recommendation was in scope.  Recommendation not in scope of 20 guidelines. |  |  |
| 33.  Health providers who provide infant feeding services, including breastfeeding support, should have sufficient knowledge, competence and skills to support women to breastfeed. Provider knowledge and skills should include:   - Breast milk production - Signs of good attachment at the breast - Effective milk transfer - How to encourage and support women with common breastfeeding problems - Appropriate resources for safe medicine use and prescribing for breastfeeding women. | **Agree: 6 guidelines** (28,35,36,42,43,49) | Agree:  1.5.6 | Agree:  43b |
|  | **Partially agree: 6 guidelines**  Of these 6 guidelines:   - 1 guideline recommended providers need knowledge of the effective signs of breastfeeding and expected frequency of feeds (20). - 4 guidelines recommended providers need knowledge of safe medicine prescribing in relation to mental health (37,40,45,48). - 1 guideline recommended maternal and child health nurses have the skills and knowledge to comply with their Program Resource Guide (38). This separate guide includes very little information of the specific breastfeeding skills/knowledge required. |  |  |
|  | **Modifications: 1 guideline**   - This 1 guideline recommended health providers undertake appropriate education to ensure safe practices when handling, labelling, and storing expressed breastmilk (29). |  |  |
|  | **Disagree: 0 guidelines** |  |  |
|  | **Absent from 3 guidelines** where the recommendation was in scope.  Recommendation not in scope of 15 guidelines. |  |  |
| 34.  Those providing breastfeeding support should:   - Be respectful of women's personal space, cultural influences, preferences and previous experience of infant feeding - Balance the woman's preference for privacy to breastfeed and express milk in hospital with the need to carry out routine observations - Obtain consent before offering physical assistance with breastfeeding - Recognise the emotional impact of breastfeeding - Give women the time, reassurance and encouragement they need to gain confidence in breastfeeding. | **Agree: 0 guidelines** | Agree:  1.5.8 | Absent |
|  | **Partially agree: 7 guidelines**  Of these 7 guidelines:   - 6 guidelines recommended those providing support should be respectful of women's personal space, cultural influences, preferences and previous experience of infant feeding (28,35,40,43,46,49). - 0 guidelines recommended those providing support should balance the woman's preference for privacy to breastfeed and express milk in hospital with the need to carry out routine observations. - 3 guidelines recommended those providing support should obtain consent before offering physical assistance with breastfeeding (20,28,35). - 5 guidelines recommended those providing support should recognise the emotional impact of breastfeeding (28,35,40,43,49). - 4 guidelines recommended those providing support should give women the time, reassurance and encouragement they need to gain confidence in breastfeeding (28,35,43,49). |  |  |
|  | **Modifications: 1 guideline**   - This 1 guideline recommended that providers should observe and be aware of concerning mother-infant interactions which may include infants experiencing serious and continued feeding problems and failing to thrive (37). |  |  |
|  | **Disagree: 0 guidelines** |  |  |
|  | **Absent from 5 guidelines** where the recommendation was in scope.  Recommendation not in scope of 18 guidelines. |  |  |
| 35.  When discussing babies' feeding:   - Acknowledge the parents' emotional, social, financial and environmental concerns about feeding options - Be respectful of parents' choices. | **Agree: 5 guidelines** (28,35,42,43,49) | Agree:  1.5.1 | Absent |
|  | **Partially agree: 5 guidelines**  Of these 5 guidelines:   - 1 guideline recommended being acknowledging women’s emotional concerns including any mental health problems when making feeding choices (40). - 1 guideline recommended being acknowledging parents’ social, financial, and environmental concerns about feeding options (50). - 1 guidelines recommended being respectful of parents’ feeding choices (32). - 2 guidelines recommended being respectful of women’s choices and preferences during all maternity care, but not specifically in relation to breastfeeding (20,38). |  |  |
|  | **Modifications: 0 guidelines** |  |  |
|  | **Disagree: 0 guidelines** |  |  |
|  | **Absent from 4 guidelines** where the recommendation was in scope.  Recommendation not in scope of 17 guidelines. |  |  |
| 36.  Before and after the birth, discuss breastfeeding and provide information and breastfeeding support (see also recommendations 37-40). Topics to discuss may include:   - Nutritional benefits for the baby - Health benefits for both the baby and the woman - How it can have benefits even if only done for a short time - How it can soothe and comfort the baby. | **Agree: 4 guidelines** (35,42,43,49) | Agree:  1.5.2 | Partially agree:  42 - Women should be counselled and provided with support for exclusive breastfeeding at each postnatal contact. |
|  | **Partially agree: 11 guidelines**  Of these 10 guidelines:   - 6 guidelines recommended providing general breastfeeding information but did not specify what this information should include (20,28,30,32,38,39). - 2 guidelines recommended providing information about the nutritional benefits for the baby (44,50). - 3 guidelines recommended providing information about the health benefits for both the baby and the woman (40,47,50). - 0 guidelines recommended providing information about how it can have benefits even if only done for a short time. - 0 guidelines recommended providing information about how it can soothe and comfort the baby. - 5 guidelines recommended providing breastfeeding support (20,26,30,38,47). |  |  |
|  | **Modifications: 0 guidelines** |  |  |
|  | **Disagree: 0 guidelines** |  |  |
|  | **Absent from 1 guideline** where the recommendation was in scope.  Recommendation not in scope of 15 guidelines. |  |  |
| 37.  Give breastfeeding care that is tailored to the woman's individual needs and provides:   - Face-to-face support - Written, digital or telephone information to supplement (but not replace) face-to-face support - Continuity of carer - Information about what to do and who to contact if she needs additional support - Information for partners about breastfeeding and how best to support breastfeeding women, taking into account the woman's preferences about the partner's involvement - Information about opportunities for peer support. | **Agree: 2 guidelines** (43,49) | Agree:  1.5.9 | Absent |
|  | **Partially agree: 10 guidelines**  Of these 10 guidelines:   - 5 guidelines recommended providing face-to-face support (28,29,35,39,50). - 6 guidelines recommended providing written, digital or telephone information to supplement face-to-face support (20,28,29,35,39,42). - 0 guidelines recommended providing continuity of carer. - 7 guidelines recommended providing information about what to do and who to contact if she needs additional support (28,35,38,39,42,47,50). - 5 guidelines recommended providing breastfeeding information for partners (28,29,35,38,42). - 6 guidelines recommended providing information about opportunities for peer support (28,35,38,42,44,50). |  |  |
|  | **Modifications: 0 guidelines** |  |  |
|  | **Disagree: 0 guidelines** |  |  |
|  | **Absent from 0 guidelines** where the recommendation was in scope.  Recommendation not in scope of 19 guidelines. |  |  |
| 38.  Make face-to-face breastfeeding support integral to the standard postnatal contacts for women who breastfeed. Continue this until breastfeeding is established and any problems have been addressed. | **Agree: 5 guidelines** (20,28,35,42,49) | Agree:  1.5.10 | Absent |
|  | **Partially agree: 2 guidelines**  Of these 2 guidelines:   - 1 guideline recommended face-to-face breastfeeding support but did not specify this continue until breastfeeding is established and any problems addressed (50). - 1 guideline recommended providing support until breastfeeding is established and problems have been addressed but did not specify this support should be face-to-face (43). |  |  |
|  | **Modifications: 0 guidelines** |  |  |
|  | **Disagree: 0 guidelines** |  |  |
|  | **Absent from 4 guidelines** where the recommendation was in scope.  Recommendation not in scope of 20 guidelines. |  |  |
| 39.  Be aware that younger women and women from a low income or disadvantaged background may need more support and encouragement to start and continue breastfeeding, and that continuity of carer is particularly important for these women. | **Agree: 3 guidelines** (35,42,43) | Agree:  1.5.11 | Absent |
|  | **Partially agree: 1 guideline**   - This 1 guideline recommended women with financial issues or with pregnancy in the teenage years may require additional support but does not specify this in relation to starting and continuing to breastfeed (46). |  |  |
|  | **Modifications: 3 guidelines**  Of these 3 guidelines:   - 1 guideline recommended counselling a woman to make an informed decision about infant feeding, suitable to her circumstances (49). - 2 guidelines recommended providing ‘enhanced’ maternal and child health services to provide extra support to women and families with identified additional or alternate needs (38,44). |  |  |
|  | **Disagree: 0 guidelines** |  |  |
|  | **Absent from 4 guidelines** where the recommendation was in scope.  Recommendation not in scope of 20 guidelines. |  |  |
| 40.  Provide information, advice and reassurance about breastfeeding, so women (and their partners) know what to expect, and when and how to seek help. Topics to discuss include:   - How milk is produced, how much is produced in the early stages, and the supply-and -demand nature of breastfeeding - Responsive breastfeeding - How often babies typically need to feed and for how long, taking into account individual variation - Feeding positions and how to help the baby attach to the breast - Signs of effective feeding so the woman knows her baby is getting enough milk (it is not possible to overfeed a breastfed baby; see also recommendation 52) - Expressing breast milk (by hand or with a breast pump) as part of breastfeeding and how it can be useful; safe storage and preparation of expressed breast milk; and the dangers of ‘prop’ feeding - Normal breast changes during pregnancy and after the birth - Pain when breastfeeding and when to seek help - Breastfeeding complications (for example, mastitis or breast abscess) and when to seek help - Strategies to manage fatigue when breastfeeding - Supplementary feeding with formula milk that is sometimes, but not commonly, clinically indicated - How breastfeeding can affect the woman’s body image and identity - That the information given may change as the baby grows - The possibility of relactation after a gap in breastfeeding - Safe medicine use when breastfeeding. | **Agree: 0 guidelines** | Agree:  1.5.12 | Partially agree:  42 - Women should be counselled and provided with support for exclusive breastfeeding at each postnatal contact. |
|  | **Partially agree: 18 guidelines**  Of these 18 guidelines:   - 6 guidelines recommended discussing how milk is produced, how much is produced in the early stages, and the supply-and -demand nature of breastfeeding (28,35,42–44,49). - 6 guidelines recommended discussing responsive breastfeeding (20,28,35,42,43,49). - 5 guidelines recommended discussing how often babies typically need to feed and for how long, taking into account individual variation (20,28,35,43,49). - 7 guidelines recommended discussing feeding positions and how to help the baby attach to the breast (20,21,28,35,42,43,49). - 5 guidelines recommended discussing signs of effective feeding so the woman knows her baby is getting enough milk (it is not possible to overfeed a breastfed baby) (28,35,42,43,49). - 6 guidelines recommended discussing expressing breast milk (by hand or with a breast pump) as part of breastfeeding and how it can be useful; safe storage and preparation of expressed breast milk (28,29,35,42,43,49). - 2 guidelines recommended discussing the dangers of ‘prop’ feeding (42,43). - 3 guidelines recommended discussing normal breast changes during pregnancy and after the birth (28,35,43). - 5 guidelines recommended discussing pain when breastfeeding and when to seek help (28,35,43,44,49). - 5 guidelines recommended discussing breastfeeding complications (for example, mastitis or breast abscess) and when to seek help (28,35,43,44,49). - 1 guideline recommended discussing strategies to manage fatigue when breastfeeding (38). - 4 guidelines recommended discussing supplementary feeding with formula milk that is sometimes, but not commonly, clinically indicated (28,35,43,49). - 2 guidelines recommended discussing how breastfeeding can affect the woman’s body image and identity (40,43). - 4 guidelines recommended discussing that the information given may change as the baby grows (28,35,42,43). - 2 guidelines recommended discussing the possibility of relactation after a gap in breastfeeding (28,35). - 9 guidelines recommended discussing safe medicine use when breastfeeding (22,28,35,37,43,45,48–50). - 3 guidelines recommended providing general breastfeeding advice but did not specify what this advice should entail (30,38,39). |  |  |
|  | **Modifications: 0 guidelines** |  |  |
|  | **Disagree: 0 guidelines** |  |  |
|  | **Absent from 1 guideline** where the recommendation was in scope.  Recommendation not in scope of 12 guidelines. |  |  |
| 41.  Give information about how the partner can support the woman to breastfeed, including:   - The value of their involvement and support - How they can comfort and bond with the baby. | **Agree: 3 guidelines** (28,35,49) | Agree:  1.5.3 | Absent |
|  | **Partially agree: 3 guidelines**  Of these 3 guidelines:   - 3 guidelines recommended providing the partner with information about how they can support the woman to breastfeed (38,42,43). - 2 guidelines recommended providing the partner with information about the value of their involvement and support (improved breastfeeding outcomes) (42,43). - 0 guidelines recommended providing the partner with information about how they can comfort and bond with the baby. |  |  |
|  | **Modifications: 1 guideline**   - This 1 guideline recommended maternal and child health services adopt ‘father-inclusive’ (partner inclusive) practices where the needs and perspectives of fathers are incorporated into the development and delivery of services. This includes bringing partners into everyday activities such as infant feeding (44). |  |  |
|  | **Disagree: 0 guidelines** |  |  |
|  | **Absent from 3 guidelines** where the recommendation was in scope.  Recommendation not in scope of 21 guidelines. |  |  |
| 42.  Encourage the woman to have early skin-to-skin contact with her baby so that breastfeeding can start when the baby and woman are ready. | **Agree: 6 guidelines** (20,28,35,42,43,49) | Agree:  1.5.7 | Absent |
|  | **Partially agree: 1 guideline**   - This 1 guidelines recommended women should be supported to have uninterrupted skin-to-skin contact with baby during perineal repair but does not talk about breastfeeding (21). |  |  |
|  | **Modifications: 1 guideline**   - This 1 guideline recommended being flexible with the timing of initial newborn examinations and assessments so as not to restrict skin-to-skin contact (32). |  |  |
|  | **Disagree: 0 guidelines** |  |  |
|  | **Absent from 4 guidelines** where the recommendation was in scope.  Recommendation not in scope of 19 guidelines. |  |  |
| 43.  A practitioner with skills and competencies in breastfeeding support should assess breastfeeding to identify and address any concerns. | **Agree: 6 guidelines** (28,35,38,39,43,49) | Agree:  1.5.13 | Partially agree:  43b - Health providers who provide infant feeding services, including breastfeeding support, should have sufficient knowledge, competence and skills to support women to breastfeed. |
|  | **Partially agree: 3 guidelines**  Of these 3 guidelines:   - 1 guidelines recommended assessing breastfeeding to identify and address any problems (20). - 1 guideline recommended clinicians should have the appropriate skills and competencies to support women to breastfeed (42). - 1 guideline recommended midwives discuss with women, consult or refer to other health providers with the appropriate skills and competencies to assess and resolve breastfeeding problems (46). |  |  |
|  | **Modifications: 0 guidelines** |  |  |
|  | **Disagree: 0 guidelines** |  |  |
|  | **Absent from 2 guidelines** where the recommendation was in scope.  Recommendation not in scope of 20 guidelines. |  |  |
| 44.  As part of the breastfeeding assessment ask about:   - Any concerns the parents have about their baby’s feeding - How often and how long the feeds are - Rhythmic sucking and audible swallowing - If the baby is content after the feed - If the baby is waking up for feeds - The baby’s weight gain or weight loss - The number of wet and dirty nappies - The condition of the woman’s breasts and nipples   Also, observe a feed within the first 24 hours after the birth, and at least 1 other feed within the first week. | **Agree: 3 guidelines** (28,35,43) | Agree:  1.5.14 | Absent |
|  | **Partially agree: 9 guidelines**  Of these 9 guidelines:   - 4 guidelines recommended asking about any concerns the parents have about their baby’s feeding (30,38,46,50). - 2 guidelines recommended asking about how often and how long the feeds are (30,39). - 2 guidelines recommended asking about rhythmic sucking and audible swallowing (20,32). - 2 guidelines recommended asking about If the baby is content after the feed (30,39). - 0 guidelines recommended asking about if the baby is waking up for feeds. - 4 guidelines recommended asking about the baby’s weight gain or weight loss (26,32,33,50). - 3 guidelines recommended asking about the number of wet and dirty nappies (32,33,39). - 1 guideline recommended asking about the condition of the woman’s breasts and nipples (46). - 2 guidelines recommended observing a feed within the first 24 hours after the birth, and at least 1 other feed within the first week (20,32). |  |  |
|  | **Modifications: 0 guidelines** |  |  |
|  | **Disagree: 0 guidelines** |  |  |
|  | **Absent from 4 guidelines** where the recommendation was in scope.  Recommendation not in scope of 15 guidelines. |  |  |
| 45.  If there are ongoing concerns, consider observing additional feeds and other actions, such as:   - Adjusting positioning and attachment to the breast - Giving expressed milk - Referring to additional support such as a lactation consultation or peer support - Assessing for tongue-tie. | **Agree: 3 guidelines** (28,35,43) | Agree:  1.5.15 | Absent |
|  | **Partially agree: 10 guidelines**  Of these 10 guidelines:   - 1 guideline recommended observing additional feeds (20). - 1 guideline recommended adjusting positioning and attachment to the breast (49). - 3 guidelines recommended giving expressed milk (26,33,49). - 9 guidelines recommended referring to additional support such as a lactation consultation or peer support (20,30,33,38,39,42,44,46,49). - 0 guidelines recommended assessing for tongue-tie. |  |  |
|  | **Modifications: 0 guidelines** |  |  |
|  | **Disagree: 0 guidelines** |  |  |
|  | **Absent from 1 guideline** where the recommendation was in scope.  Recommendation not in scope of 17 guidelines. |  |  |
| 46.  Inform women that vitamin D supplements are recommended for all breastfeeding women. | **Agree: 0 guidelines** | Agree:  1.5.4 | Absent |
|  | **Partially agree: 0 guidelines** |  |  |
|  | **Modifications: 0 guidelines** |  |  |
|  | **Disagree: 2 guidelines**  Of these 2 guidelines:   - 1 guideline recommended that vitamin supplements available to support breastfeeding are not routinely required in the presence of an adequate diet. A link to another guideline (Vitamin D status in Pregnancy practice guideline) is also provided, however this does not cover postpartum/breastfeeding supplementation (28). - 1 guideline recommended vitamin D supplementation be limited to ‘at risk’ breastfed infants of dark skinned and veiled women. This guideline also stated the vitamin D status of women and infants in Australia requires further research due to the difficulty of balancing the risks of sun exposure against the need for vitamin D synthesis (43). |  |  |
|  | **Absent from 9 guidelines** where the recommendation was in scope.  Recommendation not in scope of 20 guidelines. |  |  |
| 47.  Inform women and their partners that under the Sex Discrimination Act 1984 (Australia), women have the right to breastfeed in 'any public space'. | **Agree: 2 guidelines** (42,43) | Agree:  1.5.5 – Uses the United Kingdom’s equivalent act (Equality Act 2010). | Absent |
|  | **Partially agree: 0 guidelines** |  |  |
|  | **Modifications: 1 guideline**   - This 1 guideline recommended providing women with information about feeding away from home and breastfeeding upon returning to work (35) |  |  |
|  | **Disagree: 0 guidelines** |  |  |
|  | **Absent from 6 guidelines** where the recommendation was in scope.  Recommendation not in scope of 22 guidelines. |  |  |
| 48.  Before and after the birth, discuss formula feeding with parents who are considering or who need to formula feed, taking into account that babies may be partially formula fed alongside breastfeeding or expressed breast milk. | **Agree: 4 guidelines** (28,35,43,49) | Agree:  1.5.16 | Absent |
|  | **Partially agree: 0 guidelines** |  |  |
|  | **Modifications: 1 guideline**   - This 1 guideline recommended that when women are separated from their infants (such as when returning to paid work), they may continue breastfeeding whenever they are together. Continuation of any breastfeeding is of benefit to the woman and infant (42). |  |  |
|  | **Disagree: 0 guidelines** |  |  |
|  | **Absent from 4 guidelines** where the recommendation was in scope.  Recommendation not in scope of 22 guidelines. |  |  |
| 49.  Information about formula feeding should include:   - The differences between breast milk and formula milk - That first infant formula is the only formula milk that babies need in the first year of life, unless there are specific medical needs - How to sterilise feeding equipment and prepare formula feeds safely, including a practical demonstration if needed - For women who are trying to establish breastfeeding and considering supplementing with formula feeding, the possible effects on breastfeeding success, and how to maintain adequate milk supply while supplementing. | **Agree: 2 guidelines** (43,49) | Agree:  1.5.17 | Absent |
|  | **Partially agree: 4 guidelines**  Of these 4 guidelines:   - 1 guideline recommended providing information about the differences between breast milk and formula milk (42). - 1 guideline recommended providing information about first infant formula and how it is the only formula milk that babies need in the first year of life, unless there are specific medical needs (35). - 2 guidelines recommended providing information about how to sterilise feeding equipment and prepare formula feeds safely, including a practical demonstration if needed (20,42). - 3 guidelines recommended providing information about how supplementing with formula feeding can affect breastfeeding success, and how to maintain adequate milk supply while supplementing (28,35,42). |  |  |
|  | **Modifications: 0 guidelines** |  |  |
|  | **Disagree: 0 guidelines** |  |  |
|  | **Absent from 3 guidelines** where the recommendation was in scope.  Recommendation not in scope of 22 guidelines. |  |  |
| 50.  For parents who formula feed:   - Have a one-to-one discussion about safe formula feeding - Provide face-to-face support - Provide written, digital or telephone information to supplement (but not replace) face-to-face support. | **Agree: 2 guidelines** (43,49) | Agree:  1.5.18 | Absent |
|  | **Partially agree: 4 guidelines**  Of these 4 guidelines:   - 4 guidelines recommended having a one-to-one discussion about safe formula feeding (20,28,35,42). - 2 guidelines recommended that support should be face-to-face support (28,35). - 0 guidelines recommended providing written, digital or telephone information to supplement (but not replace) face-to-face support. |  |  |
|  | **Modifications: 0 guidelines** |  |  |
|  | **Disagree: 0 guidelines** |  |  |
|  | **Absent from 3 guidelines** where the recommendation was in scope.  Recommendation not in scope of 22 guidelines. |  |  |
| 51.  Face-to-face formula feeding support should include:   - Advice about responsive bottle feeding and help to recognise feeding cues - Offering to observe a feed - Positions for holding a baby for bottle feeding and the dangers of 'prop' feeding - Advice about how to pace bottle feeding and how to recognise signs that a baby has had enough milk (because it is possible to overfeed a formula-fed baby), and advice about ways other than feeding that can comfort and soothe the baby - How to bond with the baby when bottle feeding, through skin-to-skin contact, eye contact and the potential benefit of minimising the number of people regularly feeding the baby. | **Agree: 2 guidelines** (43,49) | Agree:  1.5.19 | Absent |
|  | **Partially agree: 3 guidelines**  Of these 3 guidelines:   - 0 guidelines recommended support should include advice about responsive bottle feeding and help to recognise feeding cues. - 0 guidelines recommended support should include offering to observe a feed - 2 guidelines recommended support should include positions for holding a baby for bottle feeding and the dangers of 'prop' feeding (42,50). - 1 guideline recommended support should include advice about how to pace bottle feeding and how to recognise signs that a baby has had enough milk (35). - 0 guidelines recommended support should include advice about ways other than feeding that can comfort and soothe the baby. - 0 guidelines recommended support should include how to bond with the baby when bottle feeding. |  |  |
|  | **Modifications: 0 guidelines** |  |  |
|  | **Disagree: 0 guidelines** |  |  |
|  | **Absent from 5 guidelines** where the recommendation was in scope.  Recommendation not in scope of 21 guidelines. |  |  |
| 52.  For parents who are thinking about supplementing breastfeeding with formula or changing from breastfeeding to formula feeding, support them to make an informed decision. | **Agree: 5 guidelines** (28,35,42,43,49) | Agree:  1.5.20 | Absent |
|  | **Partially agree: 0 guidelines** |  |  |
|  | **Modifications: 0 guidelines** |  |  |
|  | **Disagree: 0 guidelines** |  |  |
|  | **Absent from 4 guidelines** where the recommendation was in scope.  Recommendation not in scope of 22 guidelines. |  |  |
| **Additional recommendations about infant feeding found in four or more of Australia’s guidelines** | | | |
| A9.  Health facilities should facilitate rooming-in practices wherever possible and advise parents of the benefits rooming-in has for establishing feeding and learning infant cues.  As part of this, parents should also be informed of safe sleeping practices, including the recommendation for infants to sleep in their own sleep space within the same room as parents for the first 6-12 months. | **Agree: 6 guidelines**   - 6 guidelines recommended rooming in practices (28,29,35,42,43,49). - 3 guidelines recommended informing parents of safe sleeping practices (28,35,43). | Partially agree:  1.3.13 – Contains details about safe sleeping practices. This recommendation was not included in our data extraction process. | Agree, but only when looking through the details of recommendations 43a (rooming-in) and 33 (safe sleeping practices). Recommendation 33 was not included in our data extraction process. |
| A10.  The use of pacifiers, bottles and artificial teats is discouraged in the first 4-6 weeks for women trying to establish breastfeeding. Inform parents of the risks of using pacifiers including interference with establishing breastfeeding, increased risk of middle ear infections, and dental problems with prolonged use. Respect the choice of parents who do use a pacifier with their newborn and inform them of the importance of sterilisation between each use. | **Agree: 6 guidelines** (28,35,42,43,49,50) | Absent | Partially agree:  As part of the details of recommendation 43a - counsel women on the use and risks of feeding bottles, teats and pacifiers. |
| A11.  Inform women that iodine supplementation (150 micrograms per day) is recommended for all breastfeeding women. | **Agree: 4 guidelines** (35,42,43,47) | Absent | Absent |
| A12.  Health facilities and health workers should comply with the WHO’s International Code of Marketing of Breast-milk Substitutes**.** | **Agree: 4 guidelines** (28,35,43,49) | Absent | Agree, but only when looking through the details of recommendation 43a. |
| A13.  In Australia, there are very few reasons a woman should be discouraged from breastfeeding her infant temporarily or permanently. If a woman fits into one or more of the following categories, a specialist should be sought who can discuss with the woman the benefits and risks of alternate feeding options:   - Maternal illicit drug use - Maternal prescription drug use including statins - Maternal infection including Cytomegalovirus, Hepatitis B and C viruses, Herpes Simplex virus 1, Human immunodeficiency virus, Human T lymphotropic virus types 1 and 2, Tuberculosis, Syphilis (recently acquired, < 2 years), Varicella-zoster (chickenpox and shingles) | **Agree: 4 guidelines** (29,35,42,43) | Absent | Agree, but only when looking at the document *Acceptable medical reasons for use of breast-milk substitutes* (64) linked in the details of recommendation 42. |

## Recommendations: Discharge planning

| **Discharge planning recommendations** | **Number of Australian guidelines that:**   - Agree with recommendation - Partially agree with recommendation - Present a modification of recommendation - Disagree with recommendation - Recommendation is absent | **Relevant NICE guideline recommendations** | **Relevant WHO guideline recommendations** |
| --- | --- | --- | --- |
| 53.  A minimum of four postnatal care contacts is recommended.  If birth is in a health facility, the time in the health facility constitutes the first of the four recommended postnatal contacts. | **Agree: 1 guideline** (20) | Agree:  By combining recommendations 1.1.14, 1.1.15, and 1.2.7 | Agree:  44 |
|  | **Partially agree: 3 guidelines**   - 1 guideline recommended ongoing care with a GP or midwife for up to six weeks but did not specify the number of contacts (36). - 2 guidelines for maternal and child health services recommended at least four postnatal contacts after the initial contact that occurs (primarily ) in health facilities (38,39). |  |  |
|  | **Modifications: 5 guidelines**  Of these 5 guidelines:   - 1 guideline recommended women visit their GP at two and six weeks (three contacts including birthing care in a facility) (30). - 1 guideline about perinatal mental health recommended all women have an early home visit and a six-week appointment in the community. Women with symptoms or who are at high risk of poor mental health outcomes are recommended to have additional contacts (40). - 1 guideline recommended three postnatal contacts for newborns including a full examination within 48 hours of birth, and follow up assessments at 5-7 days and at 6 weeks of age (32). - 1 guideline about neonatal jaundice recommended different frequencies of postnatal contacts for newborns with different symptoms/levels of risk. Those at low risk (jaundice only above the nipple line) should be assessed within the first 24 hours and then every 2-3 days until resolved (26). - 1 guideline about neonatal jaundice recommended all babies should be examined for jaundice every 8-12 hours in the first 72 hours of life (33). |  |  |
|  | **Disagree: 0 guidelines** |  |  |
|  | **Absent from** **0 guidelines** where the recommendation was in scope.  Recommendation not in scope of 22 guidelines. |  |  |
| 54.  Care for healthy women and newborns in the health facility is recommended for at least 24 hours after vaginal birth. | **Agree: 0 guidelines** | Absent | Agree:  45 |
|  | **Partially agree: 0 guidelines** |  |  |
|  | **Modifications: 1 guideline**   - This 1 guideline recommended that the health facility length of stay will vary according to a baby’s level of risk of jaundice. Pale skinned babies with jaundice only above the nipple line may be monitored in the community after 24 hours (26). |  |  |
|  | **Disagree: 2 guidelines**   - These 2 guidelines recommended that early discharge (within 24 hours) is a possibility if:   - Parents are advised of the signs and symptoms their baby could exhibit that require urgent medical attention (32).   - The woman and baby have no complications and had a vaginal birth (discharge possible within 6 hours of birth) or had a planned caesarean (discharge possible within 24 hours of birth) (20). |  |  |
|  | **Absent from** **2 guidelines** where the recommendation was in scope.  Recommendation not in scope of 26 guidelines. |  |  |
| 55.  Before transfer from the birthing facility to community care, or before the midwife leaves after a home birth:   - Assess the woman's health - Assess the woman’s emotional well-being - Assess the woman's bladder function by measuring the volume of the first void after giving birth - Assess the baby's health (including physical inspection and observation) - If the baby has not passed meconium, advise the parents that if the baby does not do so within 24 hours of birth, they should seek advice from a healthcare professional - Make sure there is a plan for feeding the baby, which should include observing at least 1 effective feed. | **Agree: 1 guideline** (20) | Agree:  1.1.10 | Partially agree:  46 – Does not include explicit advice on when meconium has not been passed within 24 hours of birth, or to observe an effective feed. |
|  | **Partially agree: 12 guidelines**  Of these 12 guidelines:   - 1 guidelines recommended assessing the woman’s health (46). - 2 guidelines recommended assessing the woman’s emotional well-being (40,46). - 2 guidelines recommended assessing the woman's bladder function by measuring the volume of the first void after giving birth (22,23). - 2 guidelines recommended assessing the baby's health (including physical inspection and observation) (32,46). - 4 guidelines recommended assessing an aspect of the baby's health including risk of jaundice or kernicterus (26,33,43) and hip dysplasia (27). - 1 guideline recommended advising the parents that if the baby does not pass meconium within 24 hours of birth, they should seek advice from a healthcare professional (32). - 5 guidelines recommended making sure there is a plan for feeding the baby, which should include observing at least 1 effective feed (28,32,35,43,49). - 1 guideline recommended the ‘booking’ hospital is expected to provide a discharge summary of the pregnancy and birth outcome for the GP in shared care arrangements (30). |  |  |
|  | **Modifications: 1 guideline**   - This 1 guideline recommended assessing a woman’s emotional wellbeing and possible stressors such as ongoing breastfeeding problems to assist with referral in the community setting (from maternal and child health service to specialist mental health services) (40). |  |  |
|  | **Disagree: 0 guidelines** |  |  |
|  | **Absent from** **7 guidelines** where the recommendation was in scope.  Recommendation not in scope of 10 guidelines. |  |  |
| 56.  Prior to discharging women and newborns after birth from the health facility to the home, health workers should assess:   - The skills and confidence of the woman to care for herself - The skills and confidence of the parents and caregivers to care for the newborn - The home environment - Other factors that may influence the ability to provide care for the woman and the newborn in the home - Care-seeking behaviour | **Agree: 2 guidelines 2** (35,40) | Partially agree:  1.1.11 – Prior to discharge ask women about her needs, preferences, and supports available.  1.1.12 – When deciding on the timing of discharge, consider any concerns including safeguarding issues. | Agree:  46 |
|  | **Partially agree: 11 guidelines**  Of these 11 guidelines:   - 4 guidelines recommended assessing the skills and confidence of the woman to care for herself (21,31,45,48). - 6 guidelines recommended assessing the skills and confidence of the parents and caregivers to care for the newborn (20,28,32,43,48,49). - 0 guidelines recommended assessing the home environment. - 0 guidelines recommended assessing other factors that may influence the ability to provide care for the woman and the newborn in the home. - 9 guidelines recommended assessing care-seeking behaviour (20,21,26,28,31,32,42,45,48). |  |  |
|  | **Modifications: 6 guidelines**  Of these 6 guidelines:   - 3 guidelines recommended assessing the skills and confidence of the woman to care for herself in the community setting (38,39,47). - 5 guidelines recommended assessing the skills and confidence of the parents and caregivers to care for the newborn in the community setting (30,37–39,47). - 1 guideline recommended assessing the home environment in the community setting (37). - 1 guidelines recommended assessing other factors that may influence the ability to provide care for the woman and the newborn in the home in the community setting (37). - 2 guidelines recommended assessing care-seeking behaviour in the community setting (36,37). |  |  |
|  | **Disagree: 0 guidelines** |  |  |
|  | **Absent from** **6 guidelines** where the recommendation was in scope.  Recommendation not in scope of 6 guidelines. |  |  |
| 57.  Birthing facility providers should ensure that:   - The transfer of care from the birthing facility to domiciliary and/or community services is clearly communicated between healthcare professionals. - The woman or the parents are informed about the transfer of care from the birthing facility to domiciliary and/or community services. | **Agree: 9 guidelines** (20,25,30,32,36,38,40,46,48)**.** | Agree:  1.1.9 | Absent |
|  | **Partially agree: 2 guidelines**  Of these 2 guidelines:   - 1 guideline recommended the transfer of care from the birthing facility to domiciliary and/or community services is clearly communicated between healthcare professionals (26). - 1 guideline recommended the woman or the parents are informed about the transfer of care from the birthing facility to domiciliary and/or community services (22). |  |  |
|  | **Modifications: 2 guidelines**   - These 2 guidelines recommended clear communication between health professionals and with women/parents at the transfer between community based services (34,37). |  |  |
|  | **Disagree: 0 guidelines** |  |  |
|  | **Absent from** **3 guidelines** where the recommendation was in scope.  Recommendation not in scope of 15 guidelines. |  |  |
| 58.  Ensure that there is effective and prompt communication between healthcare professionals when women transfer between services, for example, from secondary to primary care, from the birthing facility to domiciliary and community services, and between community services. This should include sharing relevant information about:   - The pregnancy, birth, postnatal period and any complications - The plan of ongoing care, including any condition that needs long-term management - Problems related to previous pregnancies that may be relevant to current care - Previous or current mental health concerns - Female genital mutilation (woman or previous child) - Who has parental responsibility for the baby, if known - The woman's next of kin - Safeguarding issues - Concerns about the woman's health and care, raised by her, her partner or a healthcare professional - Concerns about the baby's health and care, raised by the parents or a healthcare professional - The baby's feeding. | **Agree: 5 guidelines** (20,30,36,40,46) | Agree:  1.1.8 | Absent |
|  | **Partially agree: 18 guidelines**  Of these 18 guidelines:   - 7 guidelines recommended sharing information about the pregnancy, birth, postnatal period and any complications (22,23,26,31,32,34,41). - 10 guidelines recommended sharing information about the plan of ongoing care (including information about planning for next birth), including any condition that needs long-term management (22,23,25,26,31,32,34,38,39,41). - 0 guidelines recommended sharing information about problems related to previous pregnancies that may be relevant to current care. - 4 guidelines recommended sharing information about previous or current mental health concerns (24,37,45,48). - 1 guideline recommended sharing information about female genital mutilation (woman or previous child) (31). - 0 guidelines recommended sharing information about who has parental responsibility for the baby, if known. - 0 guidelines recommended sharing information about the woman's next of kin. - 5 guidelines recommended sharing information about safeguarding issues (37–39,45,50). - 4 guidelines recommended sharing information about concerns about the woman's health and care, raised by her, her partner or a healthcare professional (37,38,48,50). - 7 guidelines recommended sharing information about concerns about the baby's health and care, raised by the parents or a healthcare professional (25,26,34,37–39,50). - 4 guidelines recommended sharing information about the baby's feeding (28,29,49,50). |  |  |
|  | **Modifications: 0 guidelines** |  |  |
|  | **Disagree: 0 guidelines** |  |  |
|  | **Absent from** **7 guidelines** where the recommendation was in scope.  Recommendation not in scope of 1 guideline. |  |  |
| 59.  Before transfer from the maternity unit to community care, discuss the timing of transfer to community care with the woman, and ask her about her needs, preferences and support available. | **Agree: 3 guidelines** (20,40,46) | Agree:  1.1.11 | Absent |
|  | **Partially agree: 3 guidelines**  Of these 3 guidelines:   - 1 guideline recommended making collaborative care decisions with the women but did not specifically mention this in regard to the timing of health facility discharge (45). - 1 guideline recommended assessing psychological risk factors and discussing the impact of these with the woman after birth (48). - 1 guideline recommended discussing referrals with the woman and allowing time for questions and concerns (36). |  |  |
|  | **Modifications: 4 guidelines**  Of these 4 guidelines:   - 3 guidelines recommended discussing the transfer of care between two community-based services with the woman (instead of between the maternity unit and community services) (34,37,50). - 2 guidelines recommended discussing the transfer of care for a sub-population only. For example, families of infants requiring a growth-monitoring action plan (50) or home phototherapy (26). |  |  |
|  | **Disagree: 0 guidelines** |  |  |
|  | **Absent from** **1 guideline** where the recommendation was in scope.  Recommendation not in scope of 20 guidelines. |  |  |
| 60.  When deciding on the timing of the transfer to community care, take into account the woman's preferences, the factors in recommendations 55, 56 and 59 and any concerns, including any safeguarding issues. | **Agree: 4 guidelines** (20,36,40,46) | Agree:  1.1.12 | Absent |
|  | **Partially agree: 4 guidelines**  These 4 guidelines only considered some of the factors assessed in recommendations 68, 69, and 72 when deciding on the timing of transfer to community care:   - 1 guideline considered the woman’s physical health (45). - 3 guidelines considered the woman’s emotional health (24,45,48). - 1 guideline considered the newborn’s health and feeding (26). - 0 guidelines considered the parent’s skills and confidence to look after the woman and newborn. - 0 guidelines considered the supports available to the family. - 1 guideline considered safeguarding concerns (45). |  |  |
|  | **Modifications: 1 guideline**   - This 1 guideline recommended considering the woman’s physical and mental health, and safeguarding issues when deciding on the transfer between services within the community setting (50). |  |  |
|  | **Disagree: 0 guidelines** |  |  |
|  | **Absent from 1 guideline** where the recommendation was in scope.  Recommendation not in scope of 21 guidelines. |  |  |
| 61.  Before transfer from the maternity facility to community care, or before the midwife leaves after a home birth, give women, parents and caregivers information, educational interventions, and counselling to facilitate the transition home. This should include information about:   - The postnatal period and what to expect - The importance of pelvic floor exercises - What support is available (statutory and voluntary services) - Who to contact if any concerns arise at different stages.   Educational materials, such as written/digital education booklets, pictorials for semi-literate populations and job aids should be available. | **Agree: 1 guidelines** (20) | Partially agree:  1.1.13 – Specifies information provision for women (not parents and caregivers) and does not include that different types of educational materials (pictorial, digital) should be available. | Partially agree:  47 – Information provision for women, parents, and caregivers that prepare them for discharge, including the use of different media. Does not include details of what information should include. |
|  | **Partially agree: 18 guidelines**  Of these 18 guidelines:   - 14 guidelines recommended giving women, parents and caregivers information, educational interventions and counselling to facilitate the transition home including information about self-care (21,24,35,40,45,48), perineal care (22,31), breastfeeding (28,30,35,43,49), expressing breast milk (29), newborn hip health (27), and newborn safety (30). - 8 guidelines recommended giving women, parents and caregivers information about the postnatal period and what to expect (22,24,26,32,33,40,45,48). - 2 guidelines recommended giving women information about the importance of pelvic floor exercises (21,31). - 8 guidelines recommended giving women, parents and caregivers information about what support is available (28,30,32,35,40,42,43,48). - 13 guidelines recommended giving women, parents and caregivers information about who to contact if any concerns arise at different stages (21,22,24,26,28,30–33,40,42,43,49). - 6 guidelines recommended educational materials, such as written/digital education booklets, pictorials for semi-literate populations and job aids should be available (28,29,33,35,40,43). |  |  |
|  | **Modifications: 9 guidelines**  These 9 guidelines contained components of the recommendation, but all were based in a community healthcare setting (not on discharge from the birthing facility):   - 4 guidelines recommended giving women, parents and caregivers information, educational interventions and counselling to facilitate the transition home (25,34,37,50). - 6 guidelines recommended giving women, parents and caregivers information about the postnatal period and what to expect (34,37–39,41,47). - 8 guidelines recommended giving women, parents and caregivers information about what support is available (34,36–39,41,47,50). - 7 guidelines recommended giving women, parents and caregivers information about who to contact if any concerns arise at different stages (25,34,37–39,47,50). - 5 guidelines recommended educational materials, such as written/digital education booklets, pictorials for semi-literate populations and job aids should be available (25,34,37–39). |  |  |
|  | **Disagree: 0 guidelines** |  |  |
|  | **Absent from 1 guideline** where the recommendation was in scope.  Recommendation not in scope of 2 guidelines. |  |  |
| 62.  Provision of comprehensive contraceptive information and services during postnatal care is recommended. | **Agree: 6 guidelines** (20,28,30,35,36,38) | Agree:  1.2.1 | Agree:  24 |
|  | **Partially agree: 0 guidelines** |  |  |
|  | **Modifications: 0 guidelines** |  |  |
|  | **Disagree: 0 guidelines** |  |  |
|  | **Absent from 6 guidelines** where the recommendation was in scope.  Recommendation not in scope of 19 guidelines. |  |  |

## Recommendations: Community-based care

| **Community-based recommendations** | **Number of Australian guidelines that:**   - Agree with recommendation - Partially agree with recommendation - Present a modification of recommendation - Disagree with recommendation - Recommendation is absent | **Relevant NICE guideline recommendations** | **Relevant WHO guideline recommendations** |
| --- | --- | --- | --- |
| 63.  If birth is at home, the first postnatal contact should be as early as possible within 24 hours of birth. | **Agree: 1 guideline** (20) | Modification:  1.1.14 – The first postnatal home visit by a midwife should take place within 36 hours after transfer of care from the birth facility or after a home birth. | Agree:  44 |
|  | **Partially agree: 2 guidelines**  Of these 2 guidelines:   - 1 guideline recommended an initial full and detailed examination of the newborn within the first 48 hours after birth (does not specify location of birth) (32). - 1 guideline recommended all newborn hips require examination in hospital or after home birth by a practitioner experienced in hip examination. The guideline does not specify a timeframe for the initial assessment but recommends a repeat assessment at 1-4 weeks (27). |  |  |
|  | **Modifications: 1 guideline**  This 1 guideline recommended that Queensland Health facilities offer initial postnatal contact in the first weeks after birth, but did not specifically mention arrangements for home births (36). |  |  |
|  | **Disagree: 0 guidelines** |  |  |
|  | **Absent from 3 guidelines** where the recommendation was in scope.  Recommendation not in scope of 24 guidelines. |  |  |
| 64.  After the initial postnatal contact at the birthing facility (or at home after a homebirth), at least three additional contacts are recommended for healthy women and newborns. Ideally, these contacts should be spaced out:   - Between 48 and 72 hours usually with a domiciliary midwife - Between 7 and 14 days usually with a maternal and child health nurse, - During week six after birth with either a maternal and child health nurse, GP, or obstetrician. | **Agree: 1 guidelines** (20) | Agree:  By combining recommendations 1.1.14, 1.1.15, and 1.2.7 | Agree:  44 |
|  | **Partially agree: 9 guidelines**  Of these 9 guidelines:   - 0 guidelines recommended a postnatal contact between 48 and 72 hours. - 3 guidelines recommended an early home visit but did not specify an exact timeframe (33,35,40). - 2 guidelines recommended a postnatal contact between 7 and 14 days (30,44). - 5 guidelines recommended a postnatal contact during week six after birth (24,30,40,45,48). Four of these recommendations were specifically for a mental health assessment (24,40,45,48) - 3 guidelines recommended a schedule of visits but did not specify the timing of these (33,35,36). |  |  |
|  | **Modifications: 6 guidelines**  Of these 6 guidelines:   - 3 guidelines for maternal and child health services recommended at least four postnatal contacts but at an alternate frequency (home visit in first week, community appointments at 2 weeks, 4 weeks, 8 weeks) (37–39). - 1 guideline recommended babies with complications from jaundice require close and structured follow up either over the short term (4-6 weeks for immune haemolysis) or long term (encephalopathy) (26). - 1 guideline for newborn baby assessments recommended three postnatal contacts for newborns including a full examination within 48 hours of birth, and follow up assessments at 5-7 days and at 6 weeks of age (32). - 1 guideline recommended a newborn’s hips be assessed in hospital or after home birth and then again at, 1-4 weeks, 6-8 weeks, and 6-9 months in the community with a maternal and child health nurse, GP or paediatrician (27). |  |  |
|  | **Disagree: 0 guidelines** |  |  |
|  | **Absent from 1 guideline** where the recommendation was in scope.  Recommendation not in scope of 14 guidelines. |  |  |
| 65.  Home visits during the first week after birth by skilled health personnel or a trained community health worker are recommended for the postnatal care of healthy women and newborns. The first home visit should take place within 36 hours after transfer of care from the place of birth or after a home birth.  Where home visits are not feasible or not preferred, outpatient postnatal care contacts are recommended. | **Agree: 1 guideline** (20) | Agree:  1.1.14 | Agree:  48 |
|  | **Partially agree: 10 guidelines**  Of these 10 guidelines:   - 10 guidelines recommended one or more home visits in the first week after birth (26,28,30,35,38–40,44,49,50). - 7 guidelines recommended the home visit be with a skilled health professional (midwife, maternal and child health nurse, GP) (28,30,33,38–40,49). - 0 guidelines recommended the home visit should occur within 36 hours after transfer of care from the facility. - 1 guideline recommended when home visits are not feasible or not preferred, outpatient postnatal care contacts are recommended (44). |  |  |
|  | **Modifications: 2 guidelines**  Of these 2 guidelines:   - 1 guideline recommended when jaundice is above the nipple line at discharge in pale skinned babies more than 24 hours of age, monitor clinically in the community every 2-3 days until visibly improved (26). - 1 guideline recommended a newborn’s first community contact occur at 5-7 days of age with a GP or midwife (32). |  |  |
|  | **Disagree: 0 guidelines** |  |  |
|  | **Absent from 2 guidelines** where the recommendation was in scope.  Recommendation not in scope of 16 guidelines. |  |  |
| 66.  If a woman did not receive an antenatal health visitor (midwife) visit, consider arranging an additional early postnatal health visitor visit. | **Agree: 0 guidelines** | Agree:  1.1.16 | Absent |
|  | **Partially agree: 0 guidelines** |  |  |
|  | **Modifications: 0 guidelines** |  |  |
|  | **Disagree: 0 guidelines** |  |  |
|  | **Absent from 7 guidelines** where the recommendation was in scope.  Recommendation not in scope of 24 guidelines. |  |  |
| 67.  Psychosocial and/or psychological interventions during the antenatal and postnatal period are recommended to prevent postpartum depression and anxiety. | **Agree: 13 guidelines** (20,24,30,36–40,43–45,47,48) | Agree:  1.2.2 | Agree:  19 |
|  | **Partially agree: 1 guideline**   - This 1 guideline recommended midwives consult with or refer to other health providers if a woman has a history of antenatal depression/anxiety during pregnancy or is exhibiting signs of mental illness (46). |  |  |
|  | **Modifications: 1 guideline**   - This 1 guideline recommended assessing social and emotional wellbeing in a sub-population only (victims of family violence, and women at risk of family violence) and referring to local support services (50). |  |  |
|  | **Disagree: 0 guidelines** |  |  |
|  | **Absent from 0 guidelines** where the recommendation was in scope.  Recommendation not in scope of 16 guidelines. |  |  |

## References

20. SA Maternal, Neonatal & Gynaecology Community of Practice. Postnatal Care: Routine care of the well woman and neonate [Internet]. Government of South Australia; 2021. 16 p. Available from: https://www.sahealth.sa.gov.au/wps/wcm/connect/4024bcbf-6acd-48af-bf8e-72caec419cd1/Postnatal+Care.+Routine+care+of+the+well+woman+and+neonate_PPG_v1_0.pdf?MOD=AJPERES&amp;CACHEID=ROOTWORKSPACE-4024bcbf-6acd-48af-bf8e-72caec419cd1-nNo9HgH

21. SA Maternal, Neonatal & Gynaecology Community of Practice. Perineal Care and Repair [Internet]. Government of South Australia; 2021. 20 p. Available from: https://www.sahealth.sa.gov.au/wps/wcm/connect/0eb327fb-757f-4e2f-af4f-30a7cc00b79f/Perineal+Care+and+Repair_PPG_v1_0.pdf?MOD=AJPERES&amp;CACHEID=ROOTWORKSPACE-0eb327fb-757f-4e2f-af4f-30a7cc00b79f-nPERjV6

22. SA Maternal, Neonatal & Gynaecology Community of Practice. Third and fourth degree tear management. Government of South Australia; 2018. 10 p.

23. SA Maternal, Neonatal & Gynaecology Community of Practice. Bladder management for intrapartum and postnatal women [Internet]. Government of South Australia; 2018. 12 p. Available from: https://www.sahealth.sa.gov.au/wps/wcm/connect/a013c0804ee55f67a91cadd150ce4f37/Bladder+Management+for+Intrapartum+and+Postnatal+Women_PPG_v4_0.pdf?MOD=AJPERES&amp;CACHEID=ROOTWORKSPACE-a013c0804ee55f67a91cadd150ce4f37-nKKLCJD

24. SA Maternal, Neonatal & Gynaecology Community of Practice. Anxiety and Depression in the Perinatal Period [Internet]. Government of South Australia; 2019. 11 p. Available from: https://www.sahealth.sa.gov.au/wps/wcm/connect/c7c0ccf9-b704-4411-9b47-fbf777ac0829/Anxiety+and+Depression+in+the+Perinatal+Period_PPG_v1_0.pdf?MOD=AJPERES&amp;CACHEID=ROOTWORKSPACE-c7c0ccf9-b704-4411-9b47-fbf777ac0829-nGzKLbc

25. SA Maternal, Neonatal & Gynaecology Community of Practice. Newborn Hearing Screening [Internet]. Government of South Australia; 2022. 25 p. Available from: https://www.sahealth.sa.gov.au/wps/wcm/connect/c7c5058a-bb8a-4538-a7e5-8f63b836322b/Newborn+Hearing+Screening+PPG_V_1.0.pdf?MOD=AJPERES&amp;CACHEID=ROOTWORKSPACE-c7c5058a-bb8a-4538-a7e5-8f63b836322b-o7.lIin

26. SA Maternal, Neonatal & Gynaecology Community of Practice. Neonatal Jaundice [Internet]. Government of South Australia; 2021. 18 p. Available from: https://www.sahealth.sa.gov.au/wps/wcm/connect/943332804ee50dac985f9dd150ce4f37/Neonatal+Jaundice_PPG_v2_0.pdf?MOD=AJPERES&amp;CACHEID=ROOTWORKSPACE-943332804ee50dac985f9dd150ce4f37-nHCakSA

27. SA Maternal, Neonatal & Gynaecology Community of Practice. Neonatal Hip Screening and Management of Developmental Dysplasia of the Hip [Internet]. Government of South Australia; 2017. 8 p. Available from: https://www.sahealth.sa.gov.au/wps/wcm/connect/59837e804ee50abc982e9dd150ce4f37/Neonatal+Hip+Screening+and+Management+of+Developmental+Dysplasia+of+the+Hip_PPG_v2.pdf?MOD=AJPERES&amp;CACHEID=ROOTWORKSPACE-59837e804ee50abc982e9dd150ce4f37-nGExwr3

28. SA Maternal, Neonatal & Gynaecology Community of Practice. Breastfeeding [Internet]. Government of South Australia; 2019. 26 p. Available from: https://www.sahealth.sa.gov.au/wps/wcm/connect/2bf4bd2f-5aba-4bda-8575-53317d0e6f9f/Breastfeeding_PPG_v1_0.pdf?MOD=AJPERES&amp;CACHEID=ROOTWORKSPACE-2bf4bd2f-5aba-4bda-8575-53317d0e6f9f-nL3o2Mo

29. SA Maternal, Neonatal & Gynaecology Community of Practice. Expressed breast milk safe management and administration in SA 2018 [Internet]. Government of South Australia; 2018. 14 p. Available from: https://www.sahealth.sa.gov.au/wps/wcm/connect/eca7efbb-745a-41a8-bf32-32aa199481bf/Expressed+Breast+Milk+Safe+Management+and+Administration+in+SA+2018_CD_v1.0_25.10.18.pdf?MOD=AJPERES&amp;CACHEID=ROOTWORKSPACE-eca7efbb-745a-41a8-bf32-32aa199481bf-nKMujQG

30. SA Maternal, Neonatal & Gynaecology Community of Practice. SA GP Obstetric Shared Care Protocols [Internet]. Government of South Australia; 2020. 35 p. Available from: https://www.sahealth.sa.gov.au/wps/wcm/connect/950d1700491685ff975eff9006c065a9/SA+GPOSC+Protocols_CD_v4_2.pdf?MOD=AJPERES&amp;CACHEID=ROOTWORKSPACE-950d1700491685ff975eff9006c065a9-nCflpRb

31. Queensland Clinical Guidelines. Guideline: Perineal care [Internet]. Queensland Health; 2020. 39 p. Available from: https://www.health.qld.gov.au/__data/assets/pdf_file/0022/142384/g-pericare.pdf

32. Queensland Clinical Guidelines. Guideline: Newborn baby assessment (routine) [Internet]. Queensland Health; 2021. 25 p. Available from: https://www.health.qld.gov.au/__data/assets/pdf_file/0029/141689/g-newexam.pdf

33. Queensland Clinical Guidelines. Guideline: Neonatal jaundice [Internet]. Queensland Health; 2019. 40 p. Available from: https://www.health.qld.gov.au/__data/assets/pdf_file/0018/142038/g-jaundice.pdf

34. Queensland Health. Audiology Diagnostic Assessment Protocol [Internet]. Queensland Government; 2018. 163 p. Available from: https://www.childrens.health.qld.gov.au/wp-content/uploads/PDF/healthy-hearing/hh-audiology-protocol.pdf

35. Queensland Clinical Guidelines. Guideline: Establishing breastfeeding [Internet]. Queensland Health; 2021. 31 p. Available from: https://www.health.qld.gov.au/__data/assets/pdf_file/0033/139965/g-bf.pdf

36. Queensland Clinical Guidelines. Guideline: Maternity Shared Care Operational Framework [Internet]. Queensland Health; 2021. 13 p. Available from: https://www.health.qld.gov.au/__data/assets/pdf_file/0018/143505/g-sharedcare.pdf

37. Victorian Government Department of Health and Human Services. Perinatal mental health and psychosocial assessment [Internet]. Victorian Government; 2019. 64 p. Available from: https://www.health.vic.gov.au/publications/perinatal-mental-health-and-psychosocial-assessment-practice-resource-manual-for

38. Victorian Government Department of Health and Human Services. Maternal and child health program standards [Internet]. Melbourne: Victorian Government; 2019. 62 p. Available from: https://www.health.vic.gov.au/publications/maternal-and-child-health-program-standards

39. Victorian Government Department of Health and Human Services. Maternal and child health service practice guidelines 2009 [Internet]. Melbourne: Victorian Government; 2019. 84 p. Available from: https://www.health.vic.gov.au/publications/maternal-and-child-health-service-practice-guidelines

40. Western Australian Department of Health. Perinatal and Infant Mental Health Model of Care – a framework [Internet]. Perth: Government of Western Australia; 2016. 102 p. Available from: https://ww2.health.wa.gov.au/~/media/Files/Corporate/general-documents/Mental-Health-Network/Perinatal-and-Infant-Mental-Health-Model-of-Care.pdf

41. Australian Government Department of Health. National Framework for Neonatal Hearing Screening [Internet]. 2013. 67 p. Available from: https://www.health.gov.au/sites/default/files/documents/2021/12/national-framework-for-neonatal-hearing-screening.pdf

42. National Health and Medical Research Council. Australian Dietary Guidelines [Internet]. Canberra: National Health and Medical Research Council; 2013. 226 p. Available from: https://www.eatforhealth.gov.au/sites/default/files/2022-09/n55_australian_dietary_guidelines.pdf

43. National Health and Medical Research Council. Infant Feeding Guidelines Information for Health Workers [Internet]. Canberra: National Health and Medical Research Council; 2012. 174 p. Available from: https://www.nhmrc.gov.au/about-us/publications/infant-feeding-guidelines-information-health-workers#block-views-block-file-attachments-content-block-1

44. Australian Health Ministers’ Advisory Council. National Framework for Universal Child and Family Health Services [Internet]. Australian Government Department of Health and Ageing; 2011. 70 p. Available from: https://www1.health.gov.au/internet/main/publishing.nsf/Content/4C6E476B74CC27D1CA257BF0001B0ABD/$File/NFUCFHS_National%20Framework%20for%20Universal%20Child%20and%20Family%20Health%20Services.pdf

45. The Royal Australian and New Zealand College of Obstetricians and Gynaecologists. Best Practice Statement: Mental Health Care in the Perinatal Period [Internet]. The Royal Australian and New Zealand College of Obstetricians and Gynaecologists; 2021. 22 p. Available from: https://ranzcog.edu.au/wp-content/uploads/2022/05/Mental-Health-Care-in-the-Perinatal-Period-C-Obs-48.pdf

46. Australian College of Midwives. National midwifery guidelines for consultation and referral [Internet]. 4th ed. Canberra: Australian College of Midwives; 2021. 68 p. Available from: https://ranzcog.edu.au/wp-content/uploads/2022/05/National-Midwifery-Guidelines-for-Consultation-and-Referral-4th-Edition-2021.pdf

47. Royal Australian College of General Practitioners. Guidelines for preventive activities in general practice [Internet]. 9th ed. East Melbourne: Royal Australian College of General Practitioners; 2021. 379 p. Available from: https://www.racgp.org.au/getattachment/1ad1a26f-9c8b-4e3c-b45b-3237272b3a04/Guidelines-for-preventive-activities-in-general-practice.aspx

48. Highet N, Expert Working Group and Expert Subcommittees. Mental Health Care in the Perinatal Period: Australian Clinical Practice Guideline [Internet]. Melbourne: Centre of Perinatal Excellence; 2023 [cited 2023 Jul 11]. Available from: https://www.cope.org.au/wp-content/uploads/2023/06/COPE_2023_Perinatal_Mental_Health_Practice_Guideline.pdf

49. Baby Friendly Health Initiative Australia. BFHI Australia: Maternity Facility Handbook (revised 2021) [Internet]. 2021. 77 p. Available from: https://bfhi.org.au/wp-content/uploads/2021/09/BFHI-Handbook-Maternity-Facilities-Last-Revised-Feb-2021.pdf

50. National Aboriginal Community Controlled Health Organisation, The Royal Australian and New Zealand College of General Practitioners. National guide to a preventive health assessment for Aboriginal and Torres Strait Islander people [Internet]. 3rd ed. East Melbourne; 2018. Available from: https://www.racgp.org.au/FSDEDEV/media/documents/Clinical%20Resources/Resources/National-guide-3rd-ed-Sept-2018-web.pdf

64. World Health Organization. Acceptable medical reasons for use of breast-milk substitutes. Geneva: World Health Organization; 2009.
